# Human inherited CCR2 deficiency underlies progressive polycystic lung disease

**DOI:** 10.1016/j.cell.2023.11.036

**Published:** 2023-12-28

**Authors:** Anna-Lena Neehus, Brenna Carey, Marija Landekic, Patricia Panikulam, Gail Deutsch, Masato Ogishi, Carlos A. Arango-Franco, Quentin Philippot, Mohammadreza Modaresi, Iraj Mohammadzadeh, Melissa Corcini Berndt, Darawan Rinchai, Tom Le Voyer, Jérémie Rosain, Mana Momenilandi, Marta Martin-Fernandez, Taushif Khan, Jonathan Bohlen, Ji Eun Han, Alexandre Deslys, Mathilde Bernard, Tania Gajardo-Carrasco, Camille Soudée, Corentin Le Floc’h, Mélanie Migaud, Yoann Seeleuthner, Mi-Sun Jang, Eirini Nikolouli, Simin Seyedpour, Hugues Begueret, Jean-François Emile, Pierre Le Guen, Guido Tavazzi, Costanza Natalia Julia Colombo, Federico Capra Marzani, Micol Angelini, Francesca Trespidi, Stefano Ghirardello, Nasrin Alipour, Anne Molitor, Raphael Carapito, Mohsen Mazloomrezaei, Hassan Rokni-Zadeh, Majid Changi-Ashtiani, Chantal Brouzes, Pablo Vargas, Alessandro Borghesi, Nico Lachmann, Seiamak Bahram, Bruno Crestani, Susanta Pahari, Larry S. Schlesinger, Nico Marr, Dusan Bugonovic, Stéphanie Boisson-Dupuis, Vivien Béziat, Laurent Abel, Raphael Borie, Lisa R. Young, Robin Deterding, Mohammad Shahrooei, Nima Rezaei, Nima Parvaneh, Daniel Craven, Philippe Gros, Danielle Malo, Fernando E. Sepulveda, Lawrence M. Nogee, Nathalie Aladjidi, Bruce C. Trapnell, Jean-Laurent Casanova, Jacinta Bustamante

**Affiliations:** 1 Laboratory of Human Genetics of Infectious Diseases, Necker Branch, INSERM U1163, Necker Hospital for Sick Children, Paris 75015, France; 2 Paris Cité University, Imagine Institute, Paris 75015, France; 3 Translational Pulmonary Science Center, Cincinnati Children’s Hospital Medical Center, Cincinnati, OH 45229, USA; 4 Department of Medicine, McGill University, Montreal, QC H3G 0B1, Canada; 5 Molecular Basis of Altered Immune Homeostasis, INSERM U1163, Paris Cité University, Imagine Institute, Paris 75015, France; 6 Department of Laboratory Medicine and Pathology, University of Washington, Seattle, WA 98195, USA; 7 St Giles Laboratory of Human Genetics of Infectious Diseases, Rockefeller Branch, Rockefeller University, New York, NY 10065, USA; 8 Primary Immunodeficiencies Group, Department of Microbiology and Parasitology, School of Medicine, University of Antioquia, Medellıń, Colombia; 9 Pediatric Pulmonary and Sleep Medicine Department, Children’s Medical Center, Pediatrics Center of Excellence, Tehran University of Medical Sciences, Tehran, Iran; 10 Non-communicable Pediatric Diseases Research Center, Health Research Institute, Babol University of Medical Sciences, Babol, Iran; 11 Study Center for Primary Immunodeficiencies, Necker Hospital for Sick Children, AP-HP, Paris 75015, France; 12 Center for Inborn Errors of Immunity, Icahn School, New York, NY 10029, USA; 13 Precision Immunology Institute, Icahn School, New York, NY 10029, USA; 14 Mindich Child Health and Development Institute, Icahn School, New York, NY 10029, USA; 15 Department of Pediatrics, Icahn School, New York, NY 10029, USA; 16 Department of Microbiology, Icahn School, New York, NY 10029, USA; 17 Department of Human Immunology, Sidra Medicine, Doha, Qatar; 18 Leukomotion Laboratory, Paris Cité University, INSERM UMR-S1151, CNRS UMR-S8253, Necker Hospital for Sick Children, Paris 75015, France; 19 Curie Institute, PSL Research University, CNRS, UMR144, Paris 75248, France; 20 Pierre-Gilles de Gennes Institute, PSL Research University, Paris 75005, France; 21 Department of Pediatric Pneumology, Allergology and Neonatology, Hannover Medical School, Hannover 30625, Germany; 22 Research Center for Immunodeficiencies, Tehran University of Medical Sciences, Tehran, Iran; 23 Nanomedicine Research Association (NRA), Universal Scientific Education and Research Network (USERN), Tehran, Iran; 24 Department of Pathology, Haut-Lévèque Hospital, CHU Bordeaux, Pessac 33604, France; 25 Pathology Department, Ambroise-Paré Hospital, AP-HP, Boulogne 92100, France; 26 Pulmonology Service, Bichat Hospital, AP-HP and Paris Cité University, INSERM U1152, PHERE, Paris 75018, France; 27 Department of Surgical, Pediatric, and Diagnostic Sciences, University of Pavia, Pavia 27100, Italy; 28 Anesthesia and Intensive Care, San Matteo Research Hospital, Pavia 27100, Italy; 29 Experimental Medicine, University of Pavia, Pavia 27100, Italy; 30 Neonatal Intensive Care Unit, San Matteo Research Hospital, Pavia 27100, Italy; 31 Molecular Immuno-Rheumatology Laboratory, INSERM UMR_S1109, GENOMAX Platform, Faculty of Medicine, OMICARE University, Hospital Federation, Immunology and Hematology Research Center, Research Center in Biomedicine of Strasbourg (CRBS), Federation of Translational Medicine of Strasbourg (FMTS), University of Strasbourg, Strasbourg 67081, France; 32 Interdisciplinary Thematic Institute (ITI) of Precision Medicine of Strasbourg, University of Strasbourg, Strasbourg 67081, France; 33 Immunology Laboratory, Biology Technical Platform, Biology Pole, New Civil Hospital, Strasbourg 67091, France; 34 Dr. Shahrooei Laboratory, 22 Bahman St., Ashrafi Esfahani Blvd, Tehran, Iran; 35 Department of Medical Biotechnology, Zanjan University of Medical Sciences (ZUMS), Zanjan, Iran; 36 School of Mathematics, Institute for Research in Fundamental Sciences (IPM), Tehran, Iran; 37 Laboratory of Onco-Hematology, Necker Hospital for Sick Children, Paris 75015, France; 38 School of Life Sciences, Swiss Federal Institute of Technology, Lausanne 1015, Switzerland; 39 REBIRTH - Research Center for Translational Regenerative Medicine, Hannover 30625, Germany; 40 Biomedical Research in Endstage and Obstructive Lung Disease Hannover (BREATH), Hannover 30625, Germany; 41 Cluster of Excellence RESIST (EXC 2155), Hannover Medical School, Hannover 30625, Germany; 42 Host-Pathogen Interactions and Population Health programs, Texas Biomedical Research Institute, San Antonio, TX 78227, USA; 43 College of Health and Life Sciences, Hamad Bin Khalifa University, Doha, Qatar; 44 Institute of Translational Immunology, Brandenburg Medical School, Brandenburg 14770, Germany; 45 Division of Pulmonary and Sleep Medicine, Children’s Hospital of Philadelphia, Philadelphia, PA 19104, USA; 46 Pediatric Pulmonary Medicine, Children’s Hospital Colorado, Aurora, CO 80045, USA; 47 Clinical and Diagnostic Immunology, KU Leuven, Leuven 3000, Belgium; 48 Network of Immunity to Infection, Malignancy and Autoimmunity (NIIMA), Universal Scientific Education and Research Network (USERN), Tehran, Iran; 49 Department of Immunology, Tehran University of Medical Sciences, Tehran, Iran; 50 Department of Pediatrics, Tehran University of Medical Sciences, Tehran, Iran; 51 Division of Pediatric Pulmonology, Rainbow Babies and Children’s Hospital, Cleveland, OH 44106, USA; 52 Department of Microbiology and Immunology, McGill University, Montreal, QC H3A 2B4, Canada; 53 Department of Biochemistry, McGill University, Montreal, QC H3A 2B4, Canada; 54 Department of Human Genetics, McGill University, Montreal, QC H3G 0B1, Canada; 55 Department of Pediatrics, Johns Hopkins University School of Medicine, Baltimore, MD 21205, USA; 56 Pediatric Oncology Hematology Unit, Clinical Investigation Center (CIC), Multi-theme-CIC (CICP), University Hospital Bordeaux, Bordeaux 33000, France; 57 Departments of Medicine and Pediatrics, University of Cincinnati, College of Medicine, Cincinnati, OH 45267, USA; 58 Howard Hughes Medical Institute, New York, NY 10065, USA; 59 Department of Pediatrics, Necker Hospital for Sick Children, Paris 75015, France; 60 USERN Office, Babol University of Medical Sciences, Babol, Iran; 61 Pediatric Pulmonary Disease and Sleep Medicine Research Center, Children’s Medical Center, Pediatric Center of Excellence, Tehran, University of Medical Science, Tehran, Iran; 62 The Jackson Laboratory, Farmington, CT 06032, USA; 63 Department of Pediatrics, University of Cincinnati, College of Medicine, Cincinnati, OH 45267, USA; 64 These authors contributed equally; 65 These authors contributed equally; 66 These authors contributed equally; 67 These authors contributed equally; 68 These authors contributed equally; 69 Lead contact

## Abstract

We describe a human lung disease caused by autosomal recessive, complete deficiency of the monocyte chemokine receptor C-C motif chemokine receptor 2 (CCR2). Nine children from five independent kindreds have pulmonary alveolar proteinosis (PAP), progressive polycystic lung disease, and recurrent infections, including bacillus Calmette Guérin (BCG) disease. The *CCR2* variants are homozygous in six patients and compound heterozygous in three, and all are loss-of-expression and loss-of-function. They abolish CCR2-agonist chemokine C-C motif ligand 2 (CCL-2)-stimulated Ca^2+^ signaling in and migration of monocytic cells. All patients have high blood CCL-2 levels, providing a diagnostic test for screening children with unexplained lung or mycobacterial disease. Blood myeloid and lymphoid subsets and interferon (IFN)-γ- and granulocyte-macrophage colony-stimulating factor (GM-CSF)-mediated immunity are unaffected. CCR2-deficient monocytes and alveolar macrophage-like cells have normal gene expression profiles and functions. By contrast, alveolar macrophage counts are about half. Human complete CCR2 deficiency is a genetic etiology of PAP, polycystic lung disease, and recurrent infections caused by impaired CCL2-dependent monocyte migration to the lungs and infected tissues.

## In brief

While the chemokine receptor CCR2 has been understood as a key factor in monocyte homing in mice, the study of a naturally occurring CCR2 genetic deficiency in patients suggests that, in humans, this chemokine receptor is of particular importance for the recruitment and maturation of macrophages in the lungs.

## Graphical abstract

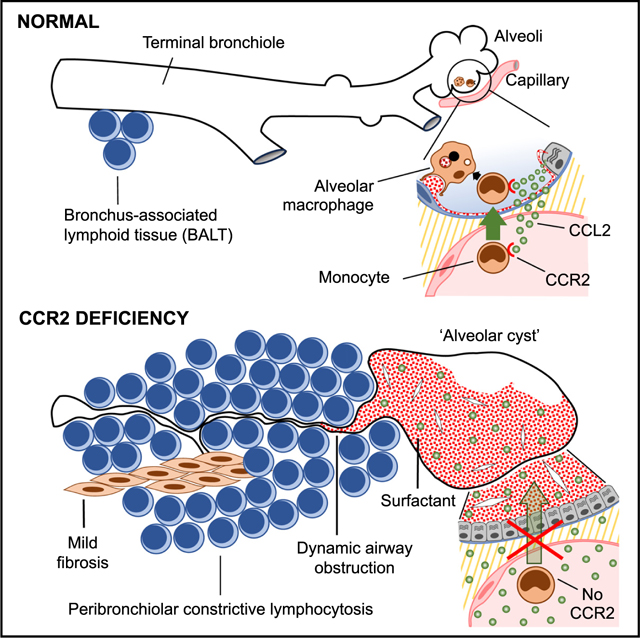

## INTRODUCTION

Pulmonary alveolar proteinosis (PAP) is a syndrome of surfactant accumulation and impaired gas exchange that occurs in various inherited and acquired diseases.^1–3^ Inherited PAP is caused by mutations in genes encoding proteins required for surfactant production (e.g., *SFTPB*, *SFTPC*, *ABCA3*, and *NKX2–1*), and depending on the gene and mutation involved, it presents at birth as respiratory failure (due to surfactant deficiency) or later in life as pulmonary fibrosis with variable degrees of surfactant accumulation (due to surfactant dysfunction).^4–6^ Inherited PAP is also caused by mutations in other genes, such as *CSF2RA* or *CSF2RB* (encoding the granulocyte-macrophage colony-stimulating factor [GM-CSF] receptor α and β chains, respectively), *STAT5B* (encoding a protein that mediates signaling by GM-CSF and other cytokines), and *GATA2*, *ADA1*, *ADA2*, *IRF8*, and *SLC7A7* (required for myeloid lineage development), which are thought to cause PAP by reducing the functions and/or numbers of alveolar macrophages.^7–13^

Acquired PAP mediated by GM-CSF-neutralizing autoantibodies is the most common etiology of PAP and accounts for 90% of all PAP patients.^1,14–18^ Alveolar macrophages require GM-CSF signaling to clear surfactant-derived cholesterol;^15,19,20^ without it, cholesterol accumulates intracellularly and is sequestered into intracytoplasmic droplets^18,21^ (a cell-protecting mechanism^22^), and the macrophages become foam cells unable to clear surfactant or to perform host defense and other functions.^23,24^ GM-CSF autoantibodies occur at low levels (<3 μg/mL serum) in people without autoimmune PAP, but high levels (>5 μg/mL serum) cause PAP.^25^ Patients with autoimmune PAP can also develop severe infectious disease due to inhaled pathogens, such as *Mycobacteria*, *Nocardia*, and *Cryptococcus*.^26–28^ PAP can also result from inhalation of toxic substances (e.g., silica, cement, or other dusts)^7,29,30^ or secondary to underlying diseases (e.g., myelodysplasia) that disrupt alveolar macrophage functions.^31^

Pediatric diffuse lung diseases are heterogeneous, typically of uncertain etiology and pathogenesis; do not have disease-specific diagnostics or therapies; and are classified on the basis of clinical, radiographic, and pathologic manifestations—i.e., as disorders of development, lung growth and alveolarization, surfactant homeostasis (i.e., PAP), and immune function; systemic disorders with pulmonary manifestations; and as disorders of uncertain pathogenesis.^32^ Disorders with immune dysfunction constitute a special group, inborn errors of immunity (IEIs), and are associated with significant morbidity and mortality.^33^ We describe a complex alveolar/interstitial/cystic lung disease associated with chronic inflammation and recurrent infections, including adverse reactions to *Mycobacterium bovis* live-attenuated bacillus Calmette Guérin (BCG) vaccine.

## RESULTS

### Nine children with pediatric progressive polycystic lung disease

**Figure 1.**
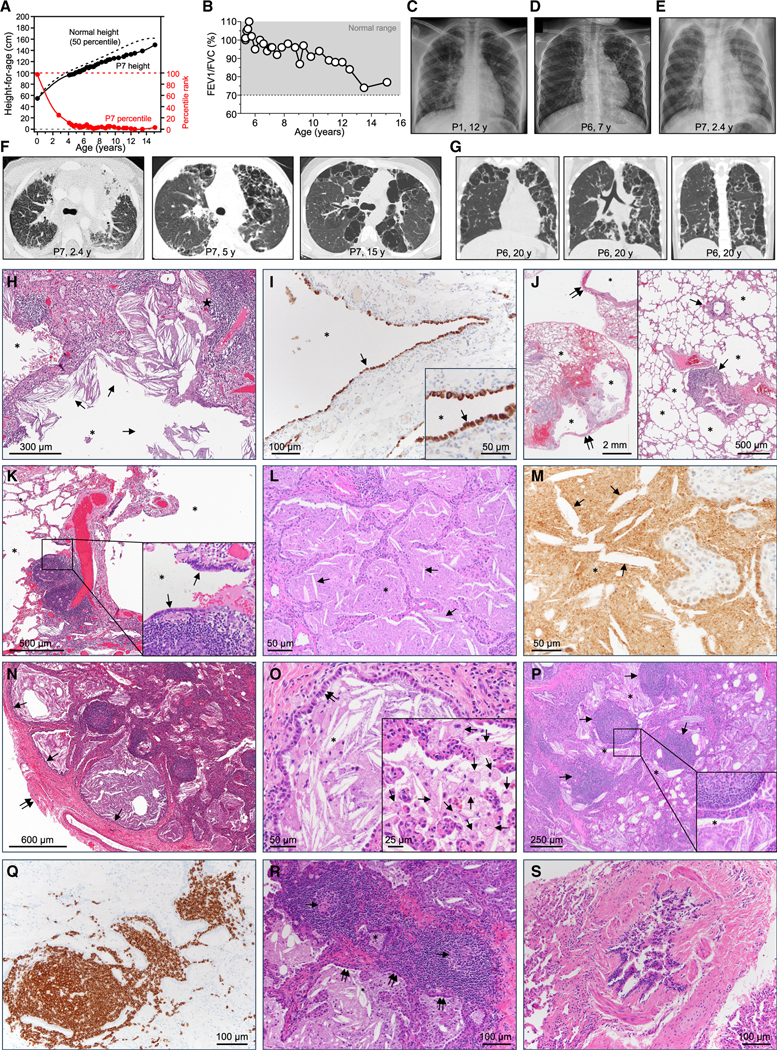
Growth curve, pulmonary function, chest radiography, and lung histology in patients with CCR2 deficiency (A) Growth curve for healthy children and P7. (B) Serial pulmonary function test results for P7. (C–E) Posterior-anterior chest X-rays. (F) Serial transverse chest CT scan images taken at the level of the carina. (G) Coronal CT scan images from the anterior chest (left), mid chest (center), and posterior chest (right). (H–K) Photomicrographs of surgical lung biopsy tissue obtained from P1 at the age of 8 years. (H) Cysts (asterisks) containing eosinophilic material, cholesterol clefts (arrows), and adjacent extensive pulmonary lymphocytosis. H&E. (I) Cysts (asterisks) lined with cuboidal epithelial cells expressing surfactant protein C (arrow). (J) Left: microscopic cysts (asterisks) located adjacent to the pleural surface (double arrows). H&E. Right: subpleural microscopic cysts (asterisks) immediately adjacent to terminal bronchioles with extensive peribronchiolar lymphocytosis (arrows) with distortion of the airway lumen. H&E. (K) Subpleural cysts (asterisks) juxtaposed to a terminal bronchiole with architectural disruption due to extensive follicular lymphocytosis (inset, arrows). H&E. (L–S) Photomicrographs of surgical lung biopsy specimen obtained from P7 at the age of 3 years. (L) Alveolae filled with eosinophilic material (asterisks) and cholesterol clefts (arrows). H&E. (M) Surfactant protein B immunostaining. (N) Cysts located immediately adjacent to the pleura and lined with cuboidal epithelium (arrows), and cysts immediately adjacent to terminal bronchioles distorted by extensive peribronchiolar follicular lymphocytic inflammation. H&E. (O) Cyst lined with cuboidal epithelial cells, containing eosinophilic material and numerous cholesterol clefts. Inset: alveolar macrophages of normal size (17.7 ± 4.2 μm, n = 29 cells). H&E. (P) Cysts (asterisks) filled with eosinophilic material and cholesterol clefts, located immediately adjacent to bronchioles displaying extensive follicular bronchiolitis. H&E. (Q) Lymphoid follicles composed of B lymphocytes. B220 immunostain. (R) Cysts lined with cuboidal epithelial cells (double arrows) immediately adjacent to terminal bronchioles obliterated (arrows) or externally compressed (asterisk) by peribronchiolar follicular bronchiolitis—constrictive cellular bronchiolitis. H&E. (S) Bronchiole with prominent subepithelial and peri-airway fibrosis. H&E. See also Figures S1 and S2.

We studied nine children with polycystic lung disease and recurrent infections; all were full term and asymptomatic at birth (Table S1, case reports 1–9). Six patients (P1, P2, and P4–P7) developed exertional dyspnea, four (P2, P4, P5, and P7) developed cough, three (P2, P6, and P7) developed growth failure, and two were asymptomatic at 12 (P3) and 15 (P8) years of age (Figure 1A; Table S1). Symptoms at presentation ranged from none to mild exertional dyspnea and occurred at a mean age of 5.1 ± 3.6 years. Physical examinations were unremarkable except for digital clubbing in four (P4–P7). Pulmonary function testing (P6–P8) revealed progressive airflow obstruction unresponsive to bronchodilator (Figures 1B and S1A). Radiographs (P1, P2, and P4–P9) identified diffuse lung disease (Figures 1C–E and S1B), and computed tomography (CT) revealed peripheral consolidation and nodular opacification; bilateral, irregular, thin-walled cysts along pleura and interlobar septa with upper lobe predominance and central sparing; and age-dependent progression in cyst number and size (Figures 1F, 1G, and S1C). Quantitative densitometry revealed cyst and parenchymal lung volumes were proportional during respiration with a constant ratio (differing by 0.5% ± 0.6% across all lobes, p = 0.98), indicating that cysts were continuous with airways (Figure S1D).

Surgical lung biopsies (P1) at 8 years of age revealed alveoli and cysts contained eosinophilic granular material and cholesterol crystals (Figure 1H), thus identifying PAP. Cysts were lined with cuboidal epithelium expressing surfactant protein C (Figure 1I) and sessile epithelium expressing aquaporin (Figure S2A), consistent with an alveolar origin. Cysts were located beneath the pleural surface (Figure 1J) and adjacent to terminal bronchioles engulfed by peribronchiolar lymphocytosis that distorted or obliterated the bronchial lumen (Figures 1H, 1J, and 1K). Surgical lung biopsies (P7) at 3 years of age revealed alveoli filled with eosinophilic granular material (Figure 1L), surfactant protein B (Figure 1M), and cholesterol crystals (Figures 1L and 1M). Microscopic cysts were present below pleural surface (Figure S2B) adjacent to terminal bronchioles that were distorted or obliterated by follicular lymphocytosis (Figure 1N) and lined with cuboidal epithelium (Figure 1O). Alveolar macrophages were not large and foamy appearing (Figure 1O, inset) as in autoimmune PAP. Alveolar type 2 epithelial cells had normal ultrastructure (Figure S2C). Follicular lymphocytosis (Figure 1P) comprised B cells (Figure 1Q) and T cells (Figure S2D) was closely associated with cysts and terminal bronchioles (Figures 1R and S2E), and distorted or obliterated the bronchiolar lumen—i.e., constrictive cellular bronchiolitis (Figure 1R). Mild peribronchiolar fibrosis (Figures 1S and S2F) and accumulation of eosinophilic material within terminal bronchioles (Figure S2E) contributed to luminal obstruction. Bronchoalveolar lavage (BAL) cytology consistently revealed low percentages of alveolar macrophages and high percentages of lymphocytes and neutrophils (Figures S2G and S2H; Table S2).

The clinical course included pustular melanosis after birth in one patient (P7), recurrent otitis media in three (P1, P2, and P7), BCG-lymphadenitis following BCG vaccination in two that resolved spontaneously (P3) or required surgical excision (P1), and disseminated BCG disease in another (P9) that responded to anti-mycobacterial therapy. P1 suffered from pneumonia (unknown cause) at the ages of 2 and 7 years; P2 presented with pneumonia at ages 4 (unknown cause), 6 (respiratory syncytial virus [RSV], rhinovirus, enterovirus, *Stenotrophomonas*, and severe acute respiratory syndrome coronavirus 2 [SARS-CoV-2]), and 8 (*Staphylococcus aureus*) years; P6 had mycoplasma pneumonia at the age of 10 years; and P7 suffered from pneumonia (unknown cause) at the age of 2 years. Eight patients (P1, P2, and P4–P9) had mild COVID-19 (Table S1). Routine laboratory tests were unremarkable, but some patients had systemic inflammation indicated by an increased erythrocyte sedimentation rate (P7), interleukin (IL)-6, or IL-8 (Figures S3A–S3F; Table S3). Five (P1–P5) had antibodies to viral or microbial pathogens, demonstrating that adaptive immune responses had occurred (Figure S3G). Serologies for toxoplasmosis (IgM and IgG) were negative in five (P1, P2, and P6–P8) patients. Serum GM-CSF autoantibody tests were negative (Figure S3H), ruling out autoimmune PAP; negative genetic testing ruled out congenital PAP (Table S1); and absence of pulmonary exposures or other underlying PAP-causing conditions ruled out secondary PAP.

### Bi-allelic rare or private *CCR2* variants

**Figure 2.**
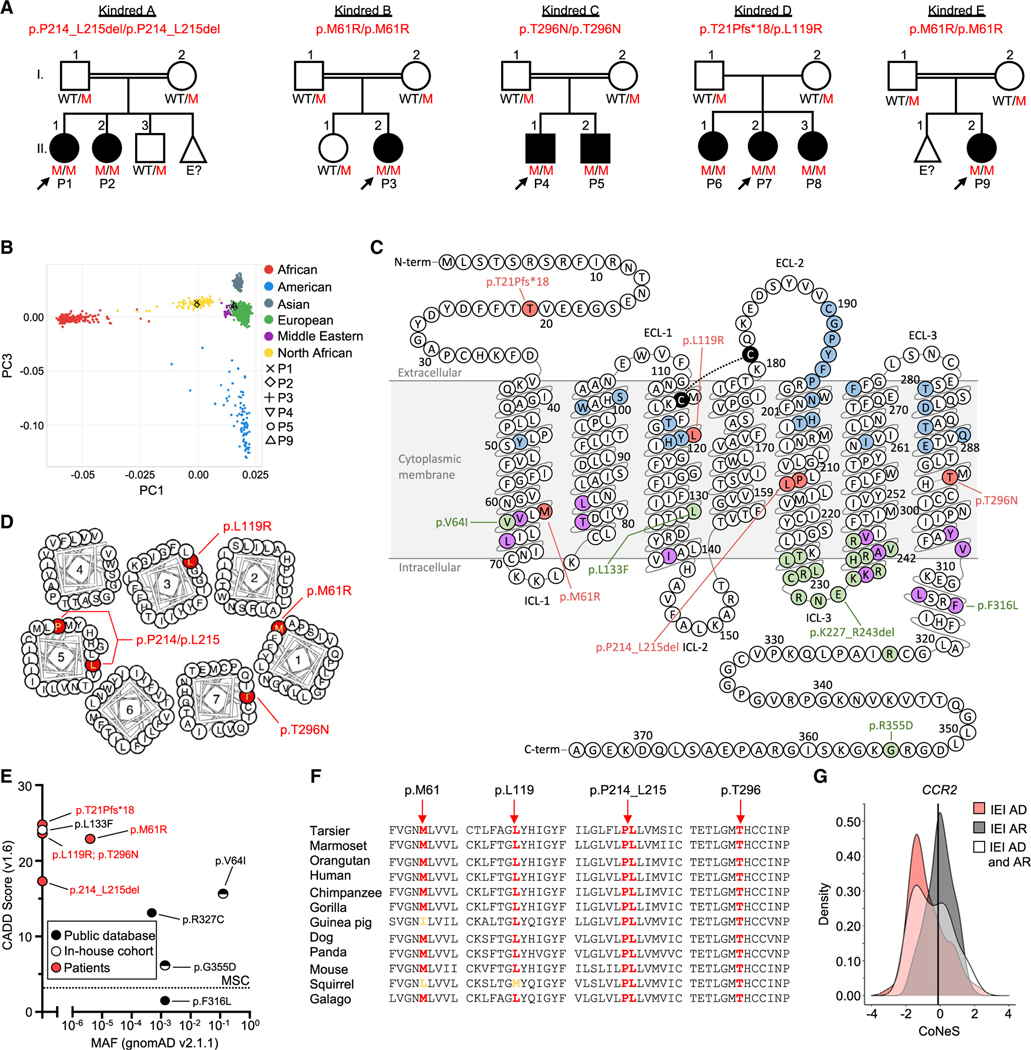
Identification, location, and evaluation of the *CCR2* variants (A) Pedigree of five unrelated kindreds. The arrow indicates the index cases. Other symbols: unknown genotype, “E?”; spontaneous abortion, triangle; consanguinity, double horizontal lines. (B) Principal-component analysis (PCA) of the WES data for the patients and samples from the 1000 Genomes database. (C) Schematic representation of the CCR2 protein. Red, patient variants; green, public database variants; blue, ligand-binding sites; purple, G protein-binding sites; black, disulfide bonds. (D) Cross-sectional schematic view of the CCR2 protein. (E) CADD MAF plot for all bi-allelic *CCR2* variants found in the patients, our in-house cohort, or in public databases. (F) Comparison of CCR2 protein sequences from diverse species. (G) Consensus negative selection (CoNeS) of *CCR2*. See also Figure S3.

The patients were of Algerian (kindred A), Iranian (kindreds B, C, and E), and American (USA, Western European) origin (kindred D) (Table S1; Figure 2A, case reports 1–9). Ancestries were confirmed by principal-component analysis (PCA) (Figure 2B) of whole-exome sequencing (WES) data (Table S4).^34^ High homozygosity rates (1.75%, 2.78%, 5.38%, 4.0%, 5.45%, and 10.2% in P1–P5 and P9, respectively) confirmed parental consanguinity in kindreds A–C and E. WES analysis focused on autosomal recessive (AR) etiologies with rare (i.e., minor allele frequency [MAF] < 0.01 in gnomAD v.2.1.1), non-synonymous variants predicted to be deleterious (i.e., combined annotation depletion-dependent [CADD] score > 99%, the mutation significance cutoff [MSC]).^35^ Six patients (P1–P5 and P9) carried homozygous rare or private C-C motif chemokine receptor 2 (*CCR2*) variants, and three (P6–P8) carried compound heterozygous private *CCR2* variants (Table S1; Figures 2C–2E). We identified no rare non-synonymous coding variants or copy-number variants of other genes, including genes underlying IEIs or PAP, common to all patients (Table S4). Sanger sequencing confirmed AR inheritance in all kindreds.

### Population genetics of the human *CCR2* locus

CCR2 is a seven-transmembrane (TM) domain G protein-coupled receptor serving as the main receptor for chemokine C-C motif ligand 2 (CCL-2), which is expressed on human monocytes, dendritic cells (DCs), and basophils.^36^ Alternative splicing results in two CCR2 isoforms (A and B), differing only in their cytoplasmic tails (Figure 2C). All patient variants altered both isoforms similarly and occurred at residues in TM domains except for p.T21Pfs*18, which caused premature termination (Figure 2C). All variants had high CADD scores and affected strongly conserved residues, suggesting they are deleterious (Figures 2E and 2F). Analysis of public databases (gnomADv2.1.1, ExAC, BRAVO/TOPMed Freeze 8, ATAV, and GME Variome; >260,000 individuals) identified four *CCR2* variants present in the homozygous state (p.V64I, affecting both CCR2 isoforms; p.F316L and p.G355D, affecting only CCR2A; p.R327C, affecting only CCR2B). One patient variant (p.M61R) with a MAF of 3.98 × 10^−6^ was found in the heterozygous state in one individual in gnomADv2.1.1. Analysis of our in-house cohort (>15,000 individuals) identified one private homozygous missense variant (p.L133F) in a patient with AR interferon (IFN)-γR2 deficiency without pulmonary manifestations.^37^ Loss-of-function (LOF) variants were infrequent in gnomADv2.1.1 (cumulative frequency of 1.49 × 10^−4^), and *CCR2* had a LOF observed-to-expected upper bound fraction ratio (LOEUF score) of 0.44, suggesting intolerance to LOF variants. The consensus negative selection (CoNeS) score of CCR2 was low (−0.11), similar to other genes associated with AR IEIs (Figure 2G).^38^

### The patients’ *CCR2* variants are loss-of-expression and LOF

**Figure 3.**
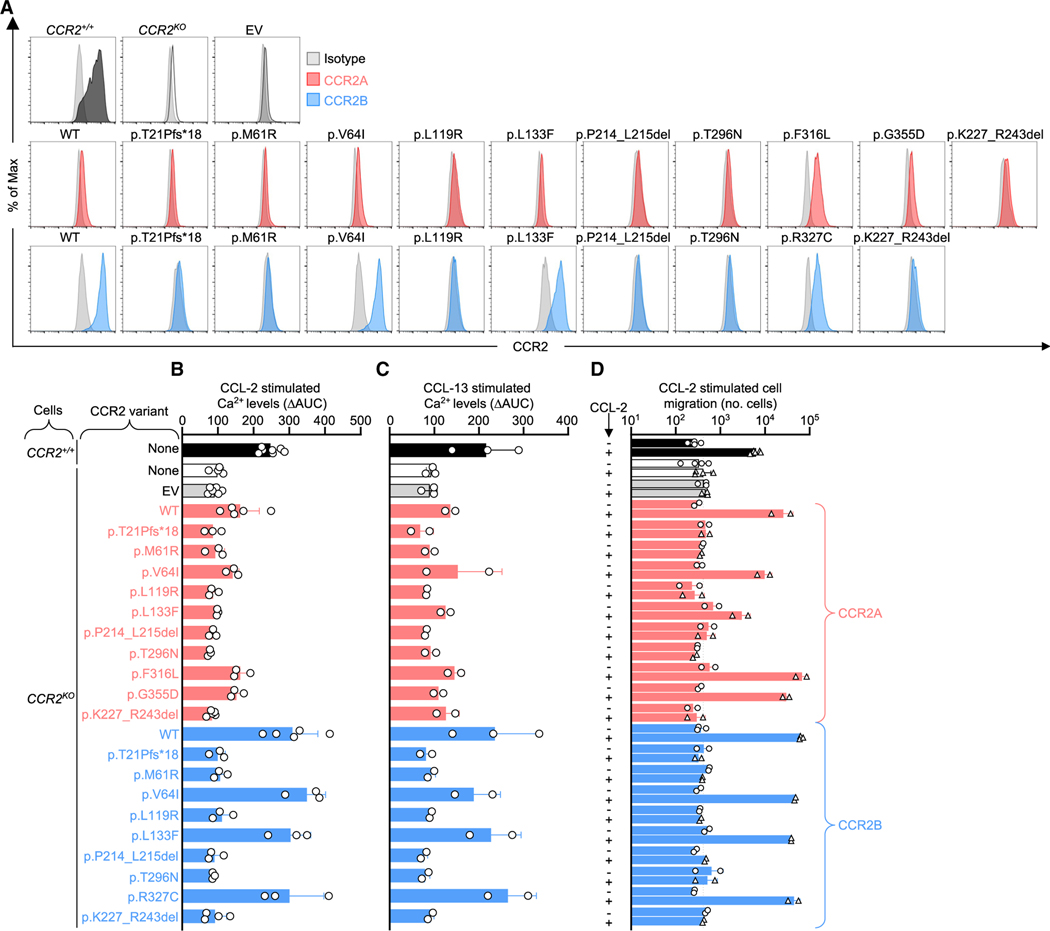
*In vitro* characterization of the *CCR2* alleles by overexpression (A) Flow cytometry with surface staining for CCR2 on THP-1 CCR2^KO^ cells transduced with an empty vector (EV) or a vector encoding the wild-type (WT) or one of the various *CCR2A* and *CCR2B* variants. The results shown are representative of three independent experiments. (B and C) Intracellular calcium (Ca^2+^) mobilization in transduced THP-1 CCR2^KO^ cells after stimulation with (B) CCL-2 or (C) ΔCCL-13 (n = 2–7 ± SD). (D) Migration of transduced THP-1 CCR2^KO^ toward CCL-2 or medium alone (n = 2–5 ± SD).

We investigated effects of *CCR2* variants on protein expression, using lentiviral transduction of CCR2-deficient (*CCR2*^KO^) THP-1 cells with wild-type (WT) or variant *CCR2*A or *CCR2B* cDNAs. No patient variants expressed CCR2A or CCR2B, whereas variants in public databases expressed both isoforms normally (Figure 3A). One public variant (p.L133F) expressed low levels of CCR2A and normal levels of CCR2B (Figure 3A). Variants were evaluated functionally by measuring Ca^2+^ mobilization and cell migration after transduction into *CCR2*^KO^ THP-1 cells. CCL-2-stimulated Ca^2+^ mobilization was rescued in cells expressing WT CCR2 but not in cells expressing variants from the patients or a functional negative control variant lacking a G protein-binding site (p.K227_R243del) (Figure 3B). By contrast, all but one homozygous variant in our in-house cohort and gnomAD rescued CCL-2-stimulated Ca^2+^ mobilization; the exception was p.L133F, which was isomorphic for CCR2B, the main isoform expressed in leukocytes,^39,40^ and hypomorphic for CCR2A (Figure 3B). CCL-13, another CCR2 ligand,^41^ did not stimulate Ca^2+^ mobilization in *CCR2*^KO^ THP-1 cells after vector-mediated expression of any patient variant (Figure 3C). By contrast, CCL-13 stimulated Ca^2+^ mobilization in *CCR2*^KO^ THP-1 cells transduced with WT *CCR2* or *CCR2* variants identified in public databases (Figure 3C). CCL2-dependent migration was also rescued in *CCR2*^KO^ THP-1 cells expressing WT *CCR2* and variants from public databases but not in cells expressing any of the patients’ variants (Figure 3D). Thus, all variants found in the patients abolish CCR2 expression and CCR2-dependent signaling. These variants are therefore LOF and result in complete CCR2 deficiency.

### The patients’ CD14^+^ monocytes do not respond to CCL-2

**Figure 4.**
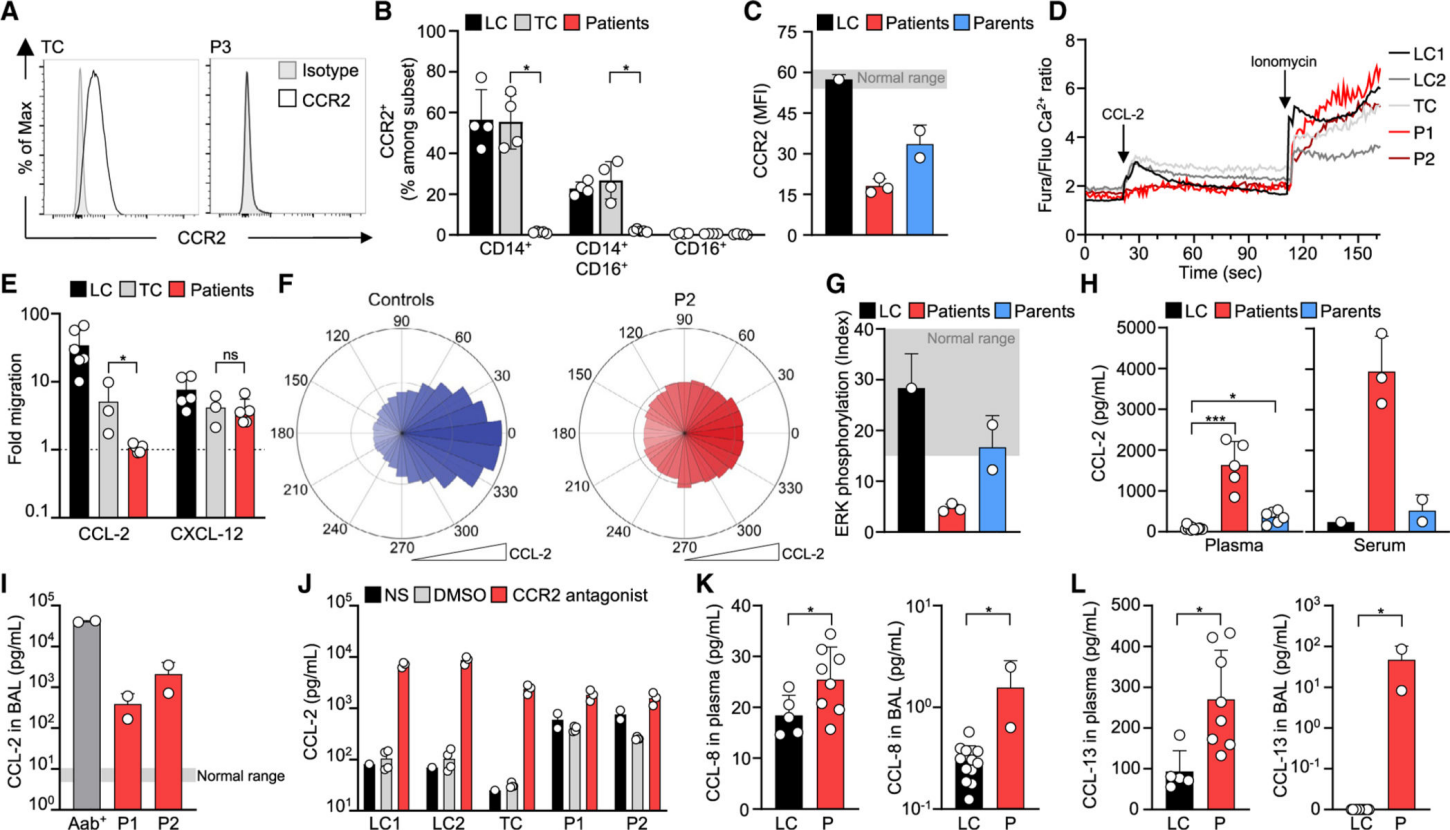
Loss of CCR2 expression and signaling in the patients’ monocytes (A and B) Representative plots (A) and calculated frequencies (B) of CCR2^+^ expression on monocyte subsets for local controls (LCs; n = 4), travel controls (TCs; n = 4), and P1–P5. (C) MFI of CCR2 staining on PBMCs from a healthy control and P6–P8 and their parents (n = 2). (D) Ca^2+^ influx assay in CD14^+^ monocytes from controls, P1, and P2 after stimulation with CCL-2 or ionomycin. (E) Fold-migration of CD14^+^ monocytes from controls (LC, n = 6; TC, n = 3) and P1–P5 toward CCL-2 or CXCL-12. (F) Distribution of trajectory angles of CD14^+^ monocytes from controls (n = 2) and P2 migrating along a CCL-2 gradient in a confined two-dimensional microenvironment. Black circles: expected angle distribution for random motion. (G) CCL-2-stimulated ERK phosphorylation in monocytes from a healthy control and P6–P8 and their parents (n = 2). (H and I) CCL-2 levels in (H) plasma or serum from healthy controls (n = 10) and P1–P8 and their parents (n = 8) or (I) in BAL relative to a patient with autoimmune PAP (Aab^+^). (J) CCl-2 levels in the supernatants of CD14^+^ monocytes from controls, P1, and P2 without stimulation (NS) or with stimulation with DMSO or a CCR2 antagonist for 24 h. (K and L) Plasma and BAL cytokine levels for CCL-8 (K) in the plasma of P1–P8 and controls (n = 5) and BAL from P1, P2, and controls (n = 12) and CCL-13 (L) in the plasma of P1–P8 and controls (n = 5) and BAL from P1, P2, and controls (n = 12). For (C), (F), (I), and (J), the data shown are the means of technical replicates ± SD; for (B), (E), (H), (K), and (L) the data shown are the means ± SD. Significance was assessed using Mann-Whitney U tests (B, E, K, and L) and Kruskal-Wallis test (H); ns, not significant; *p ≤ 0.05 and ***p ≤ 0.001. See also Figures S4 and S5.

CCR2 is strongly expressed on classical monocytes.^42^ We therefore evaluated CCR2 on peripheral blood mononuclear cells (PBMCs) and confirmed that CCR2 was abundant on classical (CD14^+^CD16^−^) monocytes and weakly present on intermediate (CD14^+^CD16^+^) monocytes, type 2 DCs, and plasmacytoid DCs (pDCs) from healthy controls (Figure S4A). CCR2 was not detected on patient monocyte subsets (P1–P5) or PBMCs (P6–P8) and was detected at intermediate levels on PBMCs from the patients’ (P6–P8) parents (Figures 4A–4C). Consistently, stimulation with CCL-2 induced Ca^2+^ mobilization in monocytes from healthy controls but not in those from patients (P1 and P2) (Figure 4D). CCL-2 also specifically stimulated the migration of monocytes from healthy controls but not monocytes from patients (P1–P5), whereas migration toward CXCL-12 was normal in both patient and control cells (Figure 4E). Live-imaging analysis demonstrated a strong impairment of the directional migration of patient monocytes (P2) toward CCL-2 relative to that observed in control monocytes (Figure 4F; Videos S1 and S2). Furthermore, PBMCs from healthy controls displayed CCL-2-stimulated phosphorylation of extracellular regulated kinase (ERK, a downstream target of CCR2 signaling^43^), whereas PBMCs from patients (P6–P8) had impaired CCL2-stimulated phosphorylation of this kinase, and PBMCs from these patients’ parents had intermediate levels of phosphorylation (Figure 4G).

### The patients have elevated CCL-2 levels

Monocytes expressing CCR2 bind, internalize, and clear CCL-2, and disruption of CCR2 receptor function increases CCL-2 levels in the blood.^44,45^ CCL-2 levels were particularly high in plasma (P1–P5), serum (P6–P8), and BAL (P1 and P2) from all patients, whereas levels were low in the plasma, serum, and BAL of healthy controls (Figures 4H, 4I, S3B, and S3D). CCL-2 levels were also higher in the culture supernatant of monocytes from patients (P1 and P2) than monocytes from healthy controls (Figure 4J). Other CCR2 ligands were present at normal (CCL-7), slightly increased (CCL-8), or high levels (CCL-13) in the patients’ plasma and BAL (Figures 4K, 4L, and S4B). Plasma or BAL concentrations of other proinflammatory cytokines or chemokines were not consistently higher in patients than in controls, except for CCL-22, pentraxin 3 (PTX3), and soluble tumor necrosis factor receptor 1 (sTNFR-RI), the levels of which were high in the patients’ BAL (Figures S3B and S4C–S4E). High IL-6 and IL-8 concentrations were occasionally detected in some patients, while CCL-2 levels were consistently high in all patients at all times evaluated (Figures S3B and S3D–S3F). CCR2-independent monocyte functions, such as the production of reactive oxygen species (ROS) and phagocytosis, were unaffected, and the expression profile of genes associated with classical monocytes was similar in patients and local controls, as demonstrated by RNA sequencing (Figures S4F–S4I). These results support the conclusion that AR complete CCR2 deficiency is the mechanism responsible for the high CCL-2 levels in the patients.

### CCR2-deficient patients have normal IFN-γ-mediated immunity

Because disruption of IFN-γ signaling causes abnormal responses to BCG,^46–48^ and three CCR2-deficient patients (P1, P3, and P9) developed lymphadenitis or disseminated BCG disease following BCG vaccination, we evaluated IFN-γ-mediated immunity (Table S1). None of the patients (P1–P5) had autoantibodies against IFN-γ (Figure S5A). STAT1 phosphorylation in response to IFN-γ was normal in all major IFN-γ-responsive leukocyte subsets (P1 and P2) tested by mass cytometry-time of flight (CyTOF), as were BCG- and IFN-γ-stimulated levels of IL-12p40 in whole blood (P1 and P2) (Figures S5B and S5C). CyTOF for IL-12- and IL-23-stimulated (both known IFN-γ inducers^49^) STAT4 phosphorylation in natural killer (NK) cells and the phosphorylation of STAT3 in mucosal-associated invariant T cells (MAIT) and γδ T cells (P1 and P2), respectively, showed levels to be similar in patients and controls (Figures S5D and S5E). IFN-γ levels in whole-blood and isolated lymphocytes (P1 and P2), following stimulation with BCG, with or without IL-12 or IL-23, were also within the normal range (Figures S5F and S5G). Lymphocyte subset analysis showed that the proportions of IFN-γ-producing innate, “innate-like” T cells, and adaptive lymphocytes were normal following stimulation with BCG, IL-12, IL-23, BCG plus IL-12, or BCG plus IL-23 (P1 and P2) (Figures S5H and S5I). There was therefore no disruption of the leukocyte-intrinsic and -extrinsic production of and responses to IFN-γ in cells from the CCR2-deficent patients *in vitro*.

### CCR2-deficient patients have normal GM-CSF-mediated signaling

Neutralizing autoantibodies against GM-CSF are the cause of disease in ~90% of patients with PAP.^1,14,17,18,23^ We therefore tested the plasma of the patients for these antibodies and found that none had an abnormal increase in the level of GM-CSF autoantibodies (Figure S3H). Consistent with this finding, the GM-CSF-neutralizing capacity of patient (P1–P5) plasma was not higher than that of control plasma (Figure S6A). GM-CSF stimulated STAT5 phosphorylation normally in control PBMCs co-cultured with plasma from patients or healthy controls but was inhibited when incubated with plasma from a patient with auto-immune PAP (Figure S6A). The patients’ (P1 and P2) monocytes displayed normal levels of CD116 (P1 and P2) and normal short-term responses to stimulation with GM-CSF or IL-3, as evaluated by assessing STAT5 phosphorylation (Figures S6B and S6C). We evaluated the response to GM-CSF further in isogenic CCR2^KO^ THP-1 cells (Figure S6D). GM-CSF-stimulated IL-8 secretion was normal in WT and CCR2^KO^ cells and was not altered by the overexpression of CCR2 (Figure S6D). Thus, human CCR2 deficiency causes pulmonary disease by a mechanism other than those previously described that involve disruption of GM-CSF signaling.

### CCR2-deficient patients have normal peripheral and bone marrow differential leukocyte counts

**Figure 5.**
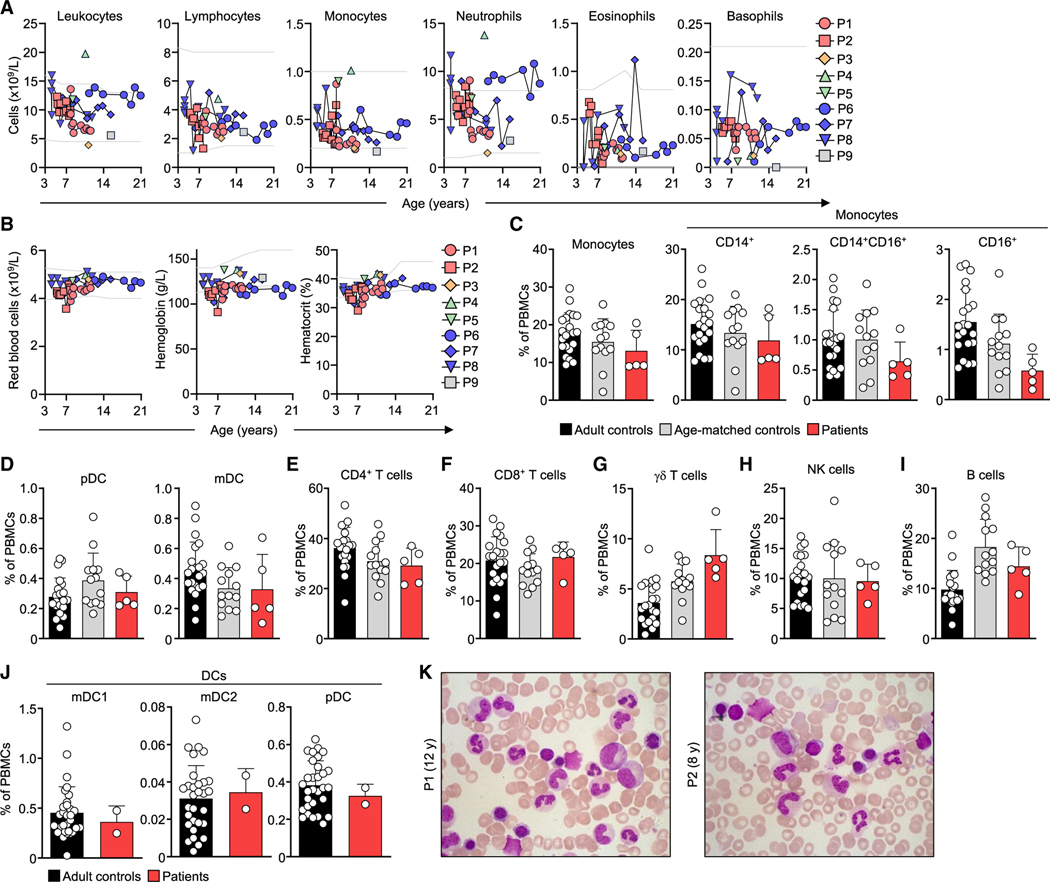
Hematological and immunological profiles of the patients with CCR2 deficiency (A and B) Absolute numbers of peripheral total (A) leukocyte subsets and (B) red blood cells, hemoglobin, and hematocrit. Gray lines represent the upper and lower limits of the normal range for each age group. (C–I) Frequency of myeloid and lymphoid subsets among PBMCs in adult controls (n = 21), age-matched controls (n = 13), and P1–P5, as determined by CyTOF. (J) Frequency of DC subsets among PBMCs in adult controls (n = 30), P1, and P2, measured by conventional flow cytometry. (K) Bone marrow aspirate smears for P1 and P2. For (C)–(J), the data shown are the mean ± SD. See also Figure S6.

*CCR2*^KO^ mice have low peripheral blood leukocyte counts due to impaired bone marrow egress.^50–52^ We therefore evaluated circulating leukocyte levels. Total blood leukocyte and differential cell counts were normal in all patients (P1–P9), as were red blood cell counts, hemoglobin concentrations, and hematocrit (Figures 5A and 5B). CyTOF evaluations of PBMCs showed that the proportions of classical and intermediate monocytes; plasmacytoid and myeloid DCs; CD4^+^, CD8^+^, and γẟ T cells; NK cells; and B cells were similar in patients (P1–P5) and adult and age-matched healthy controls (Figures 5C–5I). Detailed immunophenotyping showed that proportions of subsets of CD4^+^ and CD8^+^ T cells, NK cells, B cells, and DCs were similar in patients and age-matched healthy controls (Figures S6E–S6H). The proportions of DC subsets among PBMCs were also similar between patients and controls when assessed by conventional flow cytometry (Figure 5J). Bone marrow cytology (P1, age 12 years; P2, age 8 years) analyses showed that the proportions of granulocytic, monocytic, and lymphoid linages were normal, without clonal retraction or expansion, or malignant cells (Figure 5K; Table S5), suggesting CCR2 deficiency does not alter hematopoiesis or the egress of hematopoietic cells from the bone marrow into the bloodstream.

### Human CCR2 is expressed in myeloid precursors but not macrophages *in vitro*

We hypothesized that CCR2 might be involved in fetal monocyte homing during early hematopoiesis to establish tissue-resident macrophages in humans (as occurs in mice)^53–56^ or that it might contribute to tissue-macrophage homeostasis by recruiting adult monocytes. We used a model of primary macrophage development based on induced pluripotent stem cell (iPSC)-derived macrophages^57^ to investigate CCR2 expression over development trajectories, reproducing the early development of mononuclear phagocytes (Figures S7A–S7C). During hematopoietic differentiation *in vitro*, CCR2 expression was detected in monocytic and dendritic precursor-like cells resembling those observed at very early stages of embryonic hematopoiesis but not in hemogenic endothelial cells or in more terminally differentiated cells, such as macrophages. These data are consistent with those of previous studies^58^ showing CCR2 expression in human yolk sac-derived myeloid-biased progenitors and the monocytes derived from them, suggesting a role for CCR2 in the homing of early monocytes/precursors to tissue niches.

### Low levels of alveolar macrophages in CCR2-deficient patients

**Figure 6.**
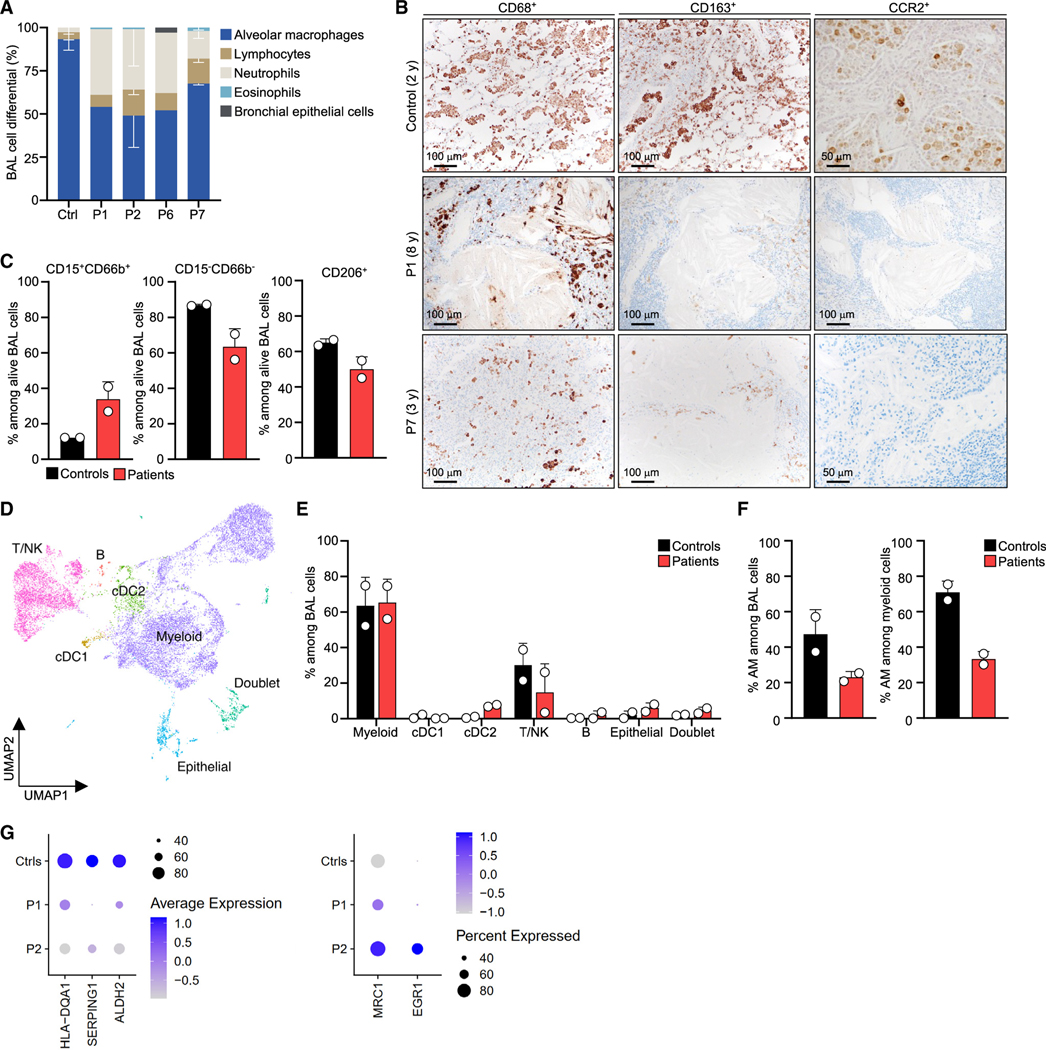
Alveolar macrophage quantification and characterization in CCR2-deficient patients (A) Cell-type composition of BAL samples from healthy reference controls^59^ and CCR2 patients taken at infection-free time points (see Table S2). (B) Immunostaining on lung biopsy tissue from P1 and P7, in comparison with a healthy control. (C) Flow cytometry analysis for the indicated cell populations on cryo-preserved BAL samples from two healthy controls, P1, and P2. (D–G) Single-cell RNA sequencing on cryo-preserved BAL samples from two healthy controls, P1, and P2 with (D) clustering analysis, (E) cell-type distribution, (F) frequency of alveolar macrophages (AMs), and (G) gene expression of selected alveolar macrophage markers within the myeloid clusters of controls and patients. For (A), (C), (E), and (F), the data shown are the mean ± SD. See also Figure S7.

PAP can be caused by a decrease in the number of alveolar macrophages.^3^ Cytological analyses of BAL obtained from four patients at times when they were free from infection (P1, P2, P6, and P7) showed that these patients had abnormally low proportions of alveolar macrophages (Figure 6A; Table S2). Both CD68^+^ and CD163^+^ alveolar macrophages were readily detected in control lung tissue but were far less abundant in lung tissues from two CCR2-deficient patients (P1 and P7) (Figures 6B and S7D). As expected, CCR2 was not detected in lung tissues from the patients (Figures 6B, S7E, and S7F). Furthermore, the alveolar macrophages observed in patients with CCR2 deficiency (P1 and P6) were not large or foamy and did not resemble those seen in patients with autoimmune PAP (Figure 1O, inset). Flow cytometry staining performed on BAL from two CCR2-deficient patients (P1 and P2) also showed the proportions of CD206^+^ alveolar macrophages to be lower than that in healthy controls (Figures 6C and S7G). Single-cell (sc) RNA sequencing of the BAL samples of these two patients revealed a normal proportion of myeloid cells in the patients’ BAL after cell-type annotation (Figures 6D and 6E). Consistent with our previous findings, the proportion of alveolar macrophage-annotated cells was lower among myeloid and total BAL cells (Figure 6F). Analysis of gene expression of 30 alveolar macrophage specific markers showed an overall normal gene expression between patients’ and controls’ myeloid cells (Figure 6G), suggesting that alveolar macrophage differentiation occurs normally in the absence of CCR2. These results support the conclusion that PAP in individuals with inherited CCR2 deficiency is caused by the presence of abnormally small numbers of alveolar macrophages due to the impaired migration of monocytes.

### Alveolar macrophages are phenotypically normal in the absence of CCR2

**Figure 7.**
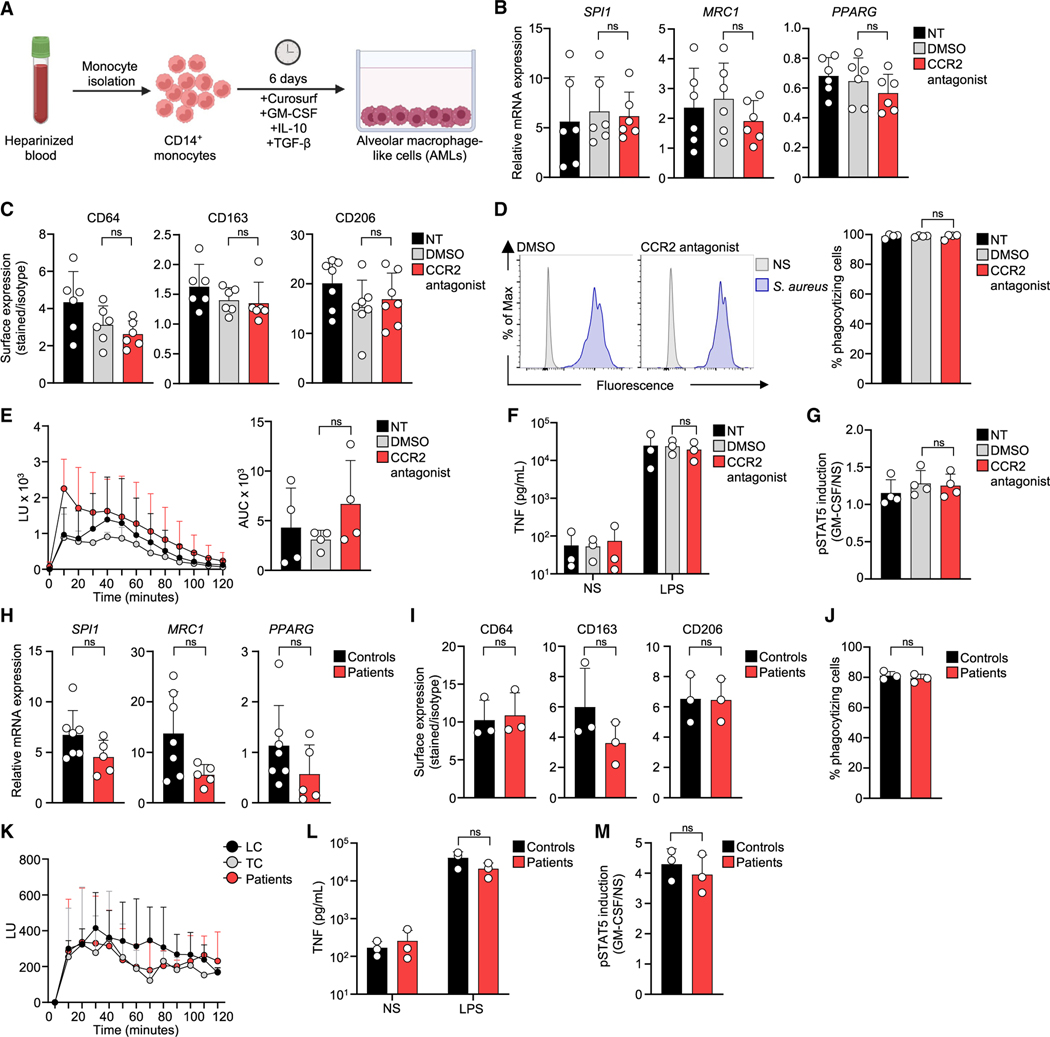
Generation of alveolar macrophage-like cells in the absence of CCR2 signaling (A) Schematic representation of the alveolar macrophage-like cell (AML) differentiation protocol. (B and C) (B) quantitative reverse-transcription PCR (RT-qPCR) and (C) flow cytometry staining on AMLs from healthy controls (n = 6) differentiated in the presence or absence (NT) of DMSO or a CCR2 antagonist. (D) Phagocytosis of pHrodo *S. aureus* bioparticles by AMLs from healthy controls (n = 4) differentiated as in (B). (E) Superoxide production in response to phorbol 12-myristate 13-acetate (PMA) by AMLs from healthy controls (n = 4) differentiated as in (B). (F) TNF secretion by AMLs from healthy controls (n = 4) differentiated as in (B) in response to LPS. (G) STAT5 phosphorylation in response to GM-CSF by AMLs from healthy controls (n = 4) as in (B). (H) RT-qPCR on AMLs from healthy controls (n = 7) and five CCR2-deficient patients (P1, P2, and P6–P8). (I) Flow cytometry surface staining on AMLs from healthy controls (n = 3) and P6–P8. (J) Phagocytosis of pHrodo *S. aureus* bioparticles by AMLs from healthy controls (n = 3) and P6–P8. (K) Superoxide production in response to PMA by AMLs from healthy controls (n = 4), P1, and P2. (L) TNF secretion by AMLs from healthy controls (n = 3) and P6–P8 in response to LPS. (M) STAT5 phosphorylation by AMLs from healthy controls (n = 3) and P6–P8 in response to GM-CSF. For (B)–(M), the data shown are the means ± SD. Significance was assessed using Mann-Whitney U tests (B–M); ns, not significant.

We further investigated the potential role of CCR2 in the differentiation of monocytes into alveolar macrophages by generating alveolar macrophage-like cells (AMLs) from healthy control monocytes in the presence or absence of a CCR2 antagonist (Figure 7A) using a recently reported method.^60^ AMLs generated under CCR2 blockade had normal levels of mRNA and phenotypic markers typical of alveolar macrophages (Figures 7B and 7C) as well as alveolar macrophage functions, including phagocytosis, ROS production, lipopolysaccharide (LPS)-stimulated proinflammatory cytokine secretion (Figures 7D–7F), and GM-CSF-stimulated STAT5 phosphorylation (Figure 7G). The differentiation of monocytes into AMLs is therefore not impaired by CCR2 signaling deficiency. We also generated AMLs from monocytes isolated from CCR2-deficient patients (P1, P2, and P6–P8) and healthy, unrelated controls. The patients’ AMLs and AMLs generated from healthy control monocytes did not differ significantly in terms of their levels of alveolar macrophage phenotypic markers (Figures 7H and 7I). The capacities of the patients’ AMLs for phagocytosis, ROS production, LPS-stimulated cytokine production, and GM-CSF-stimulated STAT5 phosphorylation were similar to those of AMLs generated from healthy controls (Figures 7J–7M). These results support the conclusion that the development of PAP in CCR2-deficient patients is due to reduced numbers of functionally normal alveolar macrophages rather than the disruption of alveolar macrophage functions.

## DISCUSSION

We describe a lung disease due to AR complete CCR2 deficiency presenting in childhood with the development of PAP, marked peribronchovascular and parenchymal lymphocytosis, with peribronchiolar pulmonary fibrosis, progressive diffuse parenchymal lung cyst formation and enlargement, progressive obstructive airflow limitation, and recurrent secondary infections. The patients are prone to clinically significant infections caused by vaccination with the weakly virulent live-attenuated BCG, thus defining a form of syndromic Mendelian Susceptibility to Mycobacterial Disease (MSMD).^61^ The variants abolish CCR2 protein expression, CCR2-stimulated Ca^2+^ mobilization, ERK phosphorylation, monocyte migration, and CCL-2 clearance, resulting in high blood CCL-2 levels, but they do not alter monocyte-specific gene expression or functions, including phagocytosis and ROS production. We detected no abnormalities of blood leukocyte counts or differential cytology, production of and signaling by GM-CSF or IFN-γ, or protective immunity to a wide range of common viral, bacterial, fungal, or parasitic pathogens, possibly partly due to the presence of intact T and B cell antigen responses and intact key functions of myeloid cells, as shown *in vitro*.

The conclusion that the patients’ CCR2 variants result in AR complete CCR2 deficiency is supported by several lines of evidence. First, all of the patients and none of their healthy relatives were homozygous or compound heterozygous for *CCR2* variants detected by exome sequencing and confirmed by Sanger sequencing; parents were heterozygous for the variants present in their respective offspring. Second, each patient CCR2 variant altered the expression of both CCR2 isoforms, disrupting CCR2 protein expression and CCR2-dependent functions, and all had high CADD scores, well above the MSC for *CCR2*,^35^ predicting deleterious effects. Third, *CCR2* had a high LOEUF score, predicting intolerance to LOF variants, and a low CoNeS score, consistent with an AR inheritance, as in other AR IEIs.^38^ Fourth, primary monocytes from patients did not respond to CCL-2 by Ca^2+^ mobilization or migration, a finding confirmed for each variant by overexpression in *CCR2*^KO^ THP-1 cells, demonstrating the loss of both expression and function. Fifth, all patients had markedly high blood levels of CCL-2, whereas their parents had intermediate levels between those of patients and unrelated healthy controls.

AR complete CCR2 deficiency increases the risk of infection likely due to reduced numbers of tissue-resident monocyte-derived macrophages since CCR2 is critical to recruit monocytes into the lungs and other tissues. In mice, CCR2^+^ monocyte recruitment replenishes alveolar macrophages during lung injury and inflammation^62,63^ and is required for lung regeneration and tracheal repair, and all these processes are impaired in CCR2-deficiency.^63,64^ Insufficient monocyte recruitment disrupts pulmonary host defense since CCR2-deficient mice are highly susceptible to lung infections by a broad spectrum of infectious agents.^65–68^ Mice overexpressing CCL-2 are protected against pulmonary *M. bovis* infection, whereas a loss of CCL-2 or CCR2 increases susceptibility to *M. tuberculosis*.^69–71^ CCR2-deficient mice also have poor recruitment of monocytes, DCs, and neutrophils to sites of mycobacterial infection and to the draining lymph nodes, impairing the priming of T cells to produce IFN-γ.^71,72^ In contrast, CCR2-deficient patients appear to have a narrower infectious phenotype restricted to pulmonary infections, recurrent otitis, and mycobacterial disease, perhaps due to the absence of the monocytopenia that occurs in CCR2-deficient mice. In CCR2-deficient patients, the egress of hematopoietic cells from the bone marrow was not impaired, whereas in CCR2-deficient mice, myeloid cells are sequestered in bone marrow, and their numbers, especially monocytes, are reduced in blood.^52^ Furthermore, human CCR2 deficiency did not alter IFN-γ production by lymphocytes, GM-CSF signaling, or myeloid host defense functions. Nevertheless, IFN-γ drives CCL-2 secretion by human neutrophils^73^ and human bronchial epithelial cells,^74^ suggesting that mycobacterial disease in patients with human CCR2 deficiency may be due to impaired CCL2-dependent monocyte recruitment to the site of infection.

The responsibility of AR complete CCR2 deficiency for mycobacterial disease and pulmonary and other infections through impairment of monocyte recruitment is supported by multiple lines of evidence, including that CCR2-deficient patients show the following: (1) recurrent pulmonary and extra-thoracic infections, (2) normal levels of blood leukocytes and their subsets, (3) normal humoral antibody immune responses, (4) normal IFN-γ-mediated immune responses, (5) normal GM-CSF signaling, (6) monocytes with a normal gene expression profile and host defense functions but no CCL-2-mediated migration, (7) small numbers of alveolar macrophages, and (8) high blood levels of CCL-2. In mice, circulating monocytes and tissue-derived CCL-2 form a homeostatic feedback loop that regulates monocyte recruitment in health and disease.^75,76^ Tissue-derived CCL-2 concentration gradients recruit monocytes to tissues, in the alveoli, for example, where they bind, internalize, and clear CCL-2, thereby reducing the concentration gradient and thus the force driving monocyte recruitment.^54,77^ Inflammation causes increased CCL-2 secretion by type II alveolar epithelial cells and neutrophils, which further increases monocyte recruitment into the lungs during infection.^51,73^ If such a mechanism also operates in humans, its disruption could account for the higher risk of infection in CCR2-deficient patients.

Histologic and cytologic observations in the CCR2-deficient patients suggest a mechanism accounting for the development of PAP: a small alveolar macrophage population unable to clear surfactant at a rate commensurate with ongoing production.^3^ The reduced numbers of alveolar macrophages in lung tissues and BAL are consistent with impaired recruitment of CCR2-deficient monocytes to the alveoli. The observation that the alveolar macrophages observed in CCR2-deficient patients were not enlarged or foamy appearing suggests that they have adequate GM-CSF-dependent cholesterol and surfactant clearance capacity, contrasting with the alveolar macrophages of humans and mice with PAP due to a disruption of GM-CSF signaling.^1,78^ The absence of high levels of GM-CSF autoantibodies and the presence of normal GM-CSF signaling in patients with CCR2 deficiency^18,22^ also rule out autoimmune PAP.^2^ None of the CCR2-deficient patients required whole-lung lavage, whereas patients with autoimmune PAP need to undergo this procedure periodically. These findings suggest that the PAP associated with CCR2 deficiency is comparatively mild but associated with prominent lymphocytosis. PAP and recurrent infections also occur in other IEIs associated with monocytopenia and deficiencies of GATA2, ADA, or IRF8.^8,9,11^

Certain histologic, radiographic, and spirometric findings suggest a mechanism explaining the ontogeny of diffuse, bilateral, peripherally located, irregularly shaped, thin-walled lung cysts, which develop during infancy or early childhood. The cysts are lined with hyperplastic cuboidal, surfactant protein C-positive epithelial cells, and sessile aquaporin-positive epithelial cells, corresponding to type II and type I alveolar epithelial cells, respectively, and contain granular, eosinophilic sediment, and cholesterol crystals—all suggesting that they originate from alveoli. The histological location of microscopic cysts below the pleural surface and the radiological location of macroscopic cysts in the lung periphery indicate that these cysts originate in the most peripheral region of the lungs. Cysts subtend terminal bronchioles surrounded by follicular lymphoid infiltrates and mild peribronchiolar fibrosis that distorts and externally compresses their lumens; terminal bronchioles also contain PAP sediment that reduces luminal patency. This constrictive cellular and fibrotic bronchiolitis probably causes dynamic “check-valve” airflow obstruction and increases intraluminal pressure in the alveoli and alveolar ducts. Such an increase in alveolar pressure during the alveolarization phase of lung growth would be expected to cause progressive alveolar enlargement resulting in, initially, microscopic and, ultimately, macroscopic parenchymal lung cysts.^79^ The synchronous dynamic changes in the volume of cysts and non-cystic lung parenchyma indicate that the lumina of the cysts are continuous with the respiratory tract. Together, these data suggest that marked constrictive peribronchiolar lymphocytosis and mild fibrosis result in dynamic obstruction of the terminal bronchioles, chronically increasing airway pressure in infancy and early childhood and leading to alveolar enlargement, which continues throughout life, resulting in an expansion of microscopic subpleural cysts into radiologically evident macroscopic, peripherally located lung cysts. The progressive increase in size suggests the luminal connection between cysts and airways is maintained over time.

Parenchymal lung cysts also occur in association with PAP caused by bi-allelic mutations in *ABCA3*^5^ and *SFTPC*^80^ and diseases associated with terminal bronchiolar obstruction such as pulmonary Langerhans cell histiocytosis.^81^ In the latter, the formation of granulomas within the terminal airway lumen results in cyst formation; interestingly, stopping smoking and therapy with cladribine, resulting in resolution of the granulomatous airway obstruction, are associated with decreases in cyst volume and resolution.^82^ Pulmonary lymphocytosis is a characteristic feature of various PAP-causing diseases, including autoimmune PAP, and function-disrupting mutations in *CSF2RA*, *CSF2RB*, and *STAT5B*.^12,13,83^ High CCL-2 levels^12,13,83–85^ are also present in these diseases, but this feature is particularly prominent in complete CCR2 deficiency. CCL-7 and CCL-8 levels are also high in autoimmune PAP,^86^ and CCL-2, −7, and −8 are all chemotactic for lymphocytes, suggesting a possible explanation for the lymphocytosis observed in CCR2 deficiency^87,88^ through a mechanism mediated by lymphocyte receptors other than CCR2, such as CCR1, CCR3, or CCR5.^77,89–91^ However, the receptors responsible for marked pulmonary lymphocyte recruitment in patients with inherited CCR2 deficiency or other PAP-causing diseases have not been identified. Further studies are required to confirm the underlying mechanisms and to characterize them more precisely.

### Limitations of the study

This study was subject to several limitations: not all the patients underwent lung biopsies; they were evaluated to differing degrees in different countries; and the precise mechanisms underlying the development and progression of the lung disease, especially follicular lymphocytosis and cyst formation and expansion, were not studied in detail in all patients. Another limitation was the use of historical rather than contemporaneous pediatric BAL controls to assess the impact of CCR2 deficiency on alveolar macrophage numbers, which could have been influenced to some degree by potential differences in the BAL technique used or in the method used for processing the BAL fluid. Nevertheless, our results demonstrate that human inherited CCR2 deficiency leads to pulmonary disease, via a mechanism including insufficient numbers of alveolar macrophages, and predisposition to mycobacterial disease.

## STAR★METHODS

### RESOURCE AVAILABILITY

#### Lead contact

Further information and requests for resources and reagents should be directed to and will be fulfilled by the lead contact, Jean-Laurent Casanova (casanova@mail.rockefeller.edu).

#### Materials availability

All raw and processed data and biological materials are available upon request from the lead contact under a Material/Data Transfer Agreement with INSERM or the Rockefeller University.

#### Data and code availability

The RNA-seq and single-cell RNA-seq data have been deposited at SRA and GEO and are publicly available as of the date of publication. Accession numbers are listed in the key resources table. The original flow cytometry data reported in this paper will be shared by the lead contact upon request. This paper does not report original code. Any additional information required to reanalyze the data reported in this paper is available from the lead contact upon request.

### EXPERIMENTAL MODEL AND STUDY PARTICIPANT DETAILS

#### Study participants

The age, sex, ancestry and race of the studied patients is reported in the main text of the paper and in the method details below. Information on gender and socioeconomic status of the patients was not collected. Seven patients were female and two were males. Written informed consent was obtained from participants or their guardians in accordance with local regulations in France, Iran and the United States of America (USA), with approval from the appropriate institutional review board (IRB). Experiments were performed in France, Qatar and the USA, in accordance with local regulations and with the approval of the IRB of INSERM and the Rockefeller University for France and the USA, respectively. The plasma samples from unrelated healthy donors used as controls for antibody profiling by PhIP-Seq were collected at Sidra Medicine in accordance with a study protocol approved by the Clinical Research Ethics Board of Sidra Medicine.

### METHOD DETAILS

#### Human patients

Informed consent was obtained in the countries of residence of the patients, in accordance with local regulations (kindred A in France, kindred B, C and E in Iran and kindred D in the USA) and with IRB approval. A detailed questionnaire covering demographic data, clinical features, and biological and microbiological results was completed by the physicians caring for the patients, and the data were sent to A-L.N. and J. Bustamante. A detailed clinical case report is provided below.

#### Case reports

**P1** is a 13-year-old girl born in 2010 to consanguineous parents originating from Algeria and living in France (**kindred A, II.1,** Table S1). She was born after a full-term pregnancy and had no respiratory abnormalities at birth. BCG vaccination resulted in local abscess formation requiring surgical excision. P1 presented recurrent cough and otitis media during early childhood, especially during the winter months. Pneumonia (without identification of the microbial pathogen responsible) occurred at the ages of two and seven years. Atopic dermatitis was diagnosed when the patient was three years old. P1 experienced a mild, self-limited infection with severe acute respiratory syndrome coronavirus 2 (SARS-CoV-2) at the age of 10 years. Serial pulmonary function testing at the ages of 9, 10, and 11 years identified progressive, mild obstructive airflow impairment (forced vital capacity (FVC) 84% the predicted value, forced expiratory flow in 1 second (FEV1) 76% the predicted value). Serial computed tomography (CT) at the ages of 9, 10, and 11 years revealed multiple thick, irregular-walled cysts, intralobular reticulation, and emphysematous change (not shown). The clinical evaluation for this study, performed at the age of 12 years, identified exertional dyspnea, severe polycystic lung disease, and allergic dermatitis (Figure 1C and data not shown). Airway bronchoscopy yielded unremarkable results. BAL yielded a fluid with an opaque, milky appearance, and cytological evaluations revealed an abnormal differential cell composition, with 54% macrophages, 38% neutrophils, 7% lymphocytes, and 1% eosinophils. BAL fluid cultures were negative for fungi, mycobacteria, and parasites and polymerase chain reaction (PCR) amplification identified no viral pathogens (Table S1). Surgical lung biopsy specimens obtained at the age of eight years revealed an abnormal accumulation of endo-alveolar eosinophilic granular sediment containing cholesterol clefts, resulting in a diagnosis of PAP. Immune studies demonstrated normal levels of IgG, IgM, and IgA and normal responses to tetanus (0.37 IU/mL [normal > 0.15 IU/mL]), diphtheria (0.53 IU/mL [normal > 0.10 IU/mL]) and pneumococcal (156.91 mg/mL [normal > 50 mg/mL]) vaccines. The patient’s parents and brother had no history of severe infection or abnormal reaction to BCG.

**P2** is an 9-year-old girl born in 2014. She is the sister of P1 (**kindred A, II.2,** Table S1). She was born after a full-term pregnancy and had no respiratory abnormalities at birth. She was vaccinated with BCG without complications. P2 suffered recurrent upper airway infections and otitis media during childhood, and recurrent pneumonia at the ages of four, six and eight years. The pathogen responsible for the bout of pneumonia at the age of four years was not identified. Multiple pathogens (respiratory syncytial virus, rhinovirus, enterovirus, *Stenotrophomonas*, and SARS-CoV-2) were implicated in the bout of pneumonia at the age of six years, and *Staphylococcus aureus* was identified during the bout of pneumonia at the age of eight years. Antibiotics and corticosteroid therapy were administered, leading to clinical resolution of the infection. A chest CT scan at the age of five years revealed diffuse polycystic lung disease and bronchiolar wall thickening in areas also displaying parenchymal consolidation. Serial pulmonary function testing identified progressive obstructive airway impairment (FVC 80% the predicted value; FEV1 57% the predicted value). Significant growth failure (height – 1 SD, weight – 2 SD) was noted at the age of seven years, leading to nasogastric tube feeding, with a clinical response. The clinical evaluation for this study, performed when the patient was eight years old, identified exertional dyspnea, severe polycystic lung disease, and allergic dermatitis. Immune studies showed that IgG, IgM, and IgA levels were normal and that the patient had normal responses to tetanus (0.34 IU/mL [normal > 0.15 IU/mL]) and diphtheria (1.85 IU/mL [normal > 0.10 IU/mL]) vaccines and a non-protective response to pneumococcal vaccine (46.87 mg/mL [normal > 50 mg/mL]).

**P3** is a 13-year-old girl born in 2010 to consanguineous parents originating from and living in Iran (**kindred B, II.2,** Table S1). She was born after a full-term pregnancy and had no respiratory abnormalities at birth. BCG vaccination resulted in BCG-itis of the local axillary lymph nodes, which healed spontaneously, without clinical sequelae. P3 developed mild allergy during infancy. The clinical evaluation for this study, performed at the age of 12 years, identified a speech disorder of unknown cause but no history of recurrent pneumonia or other infections, cough, dyspnea, or other pulmonary symptoms. The parents and sister of P3 are healthy, with no unusual infections or pulmonary diseases.

**P4** is a 13-year-old boy born in 2010 to consanguineous parents originating from and living in Iran (**kindred C, II.1,** Table S1). He was born after a full-term pregnancy and had no respiratory abnormalities at birth. He was vaccinated with BCG without complications. The patient’s medical history included frequent upper respiratory tract infections described as “colds” and mild SARS-CoV-2 infection, all of which resolved without antibiotic therapy. Clinical evaluation for this study identified a history of recurrent coughing and exertional dyspnea (when climbing one flight of stairs), and respiratory crackles noted during a physical examination at the age of 11 years. Pulmonary function testing identified obstructive airflow impairment (FVC 58.8% the predicted value). A chest X ray (Figure S1B) and CT scan (not shown) revealed diffuse ground-glass opacification and bronchiectasis. Airway bronchoscopy results were unremarkable. BAL cytology revealed an abnormal differential cell composition, with 5% macrophages, 30% neutrophils, 65% lymphocytes, a few lipid-laden macrophages, and rare hemosiderin-laden macrophages), together with the presence of Gram-positive cocci.

**P5** is a 11-year-old boy born in 2012 to consanguineous parents. He is the brother of P4 (**kindred C, II.2,** Table S1). He was born after a full-term pregnancy and had no respiratory abnormalities at birth. He was vaccinated with BCG without complications. His medical history included frequent upper respiratory tract infections described as “colds” and mild SARS-CoV-2 infection (at the age of 10 years), all of which resolved without antibiotic therapy. The clinical evaluation for this study, performed at the age of 10 years, identified a history of increased infrequent coughing, exertional dyspnea (when climbing one flight of stairs), and respiratory crackles noted during a physical examination at the age of nine years. Pulmonary function testing identified obstructive airflow impairment (FVC 74.2% the predicted value). A chest X ray (Figure S1B) and CT scan (not shown) revealed diffuse ground-glass opacification and bronchiectasis.

**P6** is a 22-year-old woman who was born to non-consanguineous parents living in the USA in 2001 (**kindred D, II.2,** Table S1). She was born after a full-term pregnancy without respiratory symptoms. She was not vaccinated with BCG. She was initially evaluated at the age of seven years, after her younger sister (P7) was found to have lung disease (see below). At presentation, P6 was asymptomatic but had mild digital clubbing and respiratory crackles on examination. Serial chest CT scans documented the presence of multiple pulmonary cysts, which increased in number and size over time and were accompanied by fibrosis and atelectasis (Figure 1G). Serial pulmonary function tests demonstrated progressive obstructive airflow impairment (Figure S1A). P6 had mycoplasma pneumonia at the age of 10 years and a mild, self-limited SARS-CoV-2 infection at the age of 20 years. The clinical evaluation for this study, performed at the age of 21 years, revealed exertional dyspnea. Immunological studies showed the patient to have normal immunoglobulin levels and chronic increases in erythrocyte sedimentation rate (ESR) and C-reactive protein concentration. Both parents are healthy, with no family history of chronic lung disease.

**P7** is a 17-year-old girl born in 2006 to non-consanguineous parents (**kindred D, II.1,** Table S1) and the sister of P6 and P8. She was the product of a full-term pregnancy without respiratory symptoms at birth. She was not vaccinated with BCG. Pustular melanosis was diagnosed at the age of three days. Although described as asymptomatic, by two years of age, she had received many courses of antibiotics for respiratory infections but had not had any steroids or any asthma therapy. At birth, the length and weight were the 97^th^ and 93^rd^ percentile but by 38 months, both had fallen to the 25^th^ percentile and continued declining thereafter (Figure 1A); gastric tube feeding was instituted. Severe digital clubbing was noticed incidentally at two years (Table S1) prompting referral and extensive evaluation. Radiologic assessment identified normal airway, vascular, and parenchymal lung lobe anatomy and the absence of bronchiectasis, pericardial or pleural effusions, vascular filling defects (Figure 1E) but did identify small axillary lymph nodes bilaterally, enlarged an enlarged pre-tracheal lymph node, and prominent bilateral hilar lymphoid tissue, likely reactive, left upper lobe nodular consolidation, and in subsequent chest CT scans numerous subpleural and peripheral parenchymal lung cysts with an upper lobe and peripheral prominence and central sparing, which increased in number and size with age (Figure 1F). A sweat chloride test was negative. Echocardiography, bronchoscopy with BAL, upper endoscopy and colonoscopy were unremarkable. At age three, surgical lung biopsies obtained from the superior segment of the left lower lobe and the left upper lobe were essentially identical and showed patchy chronic subsegmental follicular bronchiolitis with numerous large germinal centers involving greater than 50% of the parenchyma with alveolar distension by proteinaceous debris and cholesterol clefts (Figures 1L–1S). Adjacent alveolar spaces were lined by hypertrophic type II pneumocytes and markedly distended by weakly PAS-positive, nonpolarizable proteinaceous debris showing marked cholesterol clefts. Only rare PAS-positive alveolar macrophages are noted in the alveolar lumens. No exogenous food particles or lipid laden macrophages suggestive of aspiration were noted. Germinal centers show normal lymphoid architecture and EBER-1 in situ hybridization is negative for EBV. Parenchyma adjacent to the affected areas shows interstitial fibrosis and type II pneumocytes hyperplasia. Areas away from the affected regions are essentially normal in terms of alveolar structure and branching, bronchiolar and arteriolar anatomy, and septal venous architecture. Pleura was unremarkable. Iron stain showed only rare hemosiderin laden macrophages. GMS and AFB stains for fungal and mycobacteria were negative. Electron microscopy revealed normal lamellar bodies (Figure S2C) making surfactant deficiency unlikely.

At age 5, the patient underwent a thorough reassessment at Denver Children’s Hospital. The history included minor environmental exposure to chickens and mold, and allergies to grass, and pollen and mild, microcytic, hypochromic anemia and an elevated sedimentation rate and C-reactive protein were identified. Routine laboratory studies and special serologic, immunologic, and rheumatological studies were normal. Genetic studies ruled out mutations in *NKX2.1*, *SFTPC*, *SFTPB*, and a GM-CSF autoantibody test was negative (Figure S3H). Bronchoscopy with BAL was repeated and revealed a cell differential including 68 macrophages, 16 lymphocytes, 13 neutrophils, and 3 eosinophils with lipophages (index = 36) but no siderophages (iron index=0), normal flora (Gram stain) and no fungal (GMS stain) or mycobacterial (AFB) microorganisms identified. Later in childhood, chronic intermittent cough and mild exertional dyspnea developed. At 15 years, mild, self-limited SARS-CoV-2 infection occurred. At age 16, the clinical evaluation for this study identified intermittent cough, exertional dyspnea, digital clubbing, allergic dermatitis, severe polycystic lung disease, progressive, obstructive airflow limitation without bronchodilator response and a high residual volume.

**P8** is a 14-year-old girl born in 2008 to non-consanguineous parents. She is the youngest of three affected sisters (**kindred D, II.3,** Table S1). She was born after a full-term pregnancy without respiratory symptoms. She was not vaccinated with BCG. She was initially evaluated at the age of 19 months and found to have polycystic lung disease following the diagnosis of her older sister (P7; see above). She was diagnosed with allergies (to cats, dust mites, *Cladosporium*, grass and tree pollen) in early childhood, and had mild, self-limited COVID-19 at the age of 13 years. She has had no pulmonary symptoms since the age of five years. Serial pulmonary function tests show a normal FEV1/FVC ratio and borderline FEV1 suggestive of early or very slowly progressing obstructive airflow impairment (Figure S1A) with normal peripheral arterial oxygen saturation (not shown). Serial chest CT scans show an increase in the number and size of bilateral, thin- and smooth-walled lung cysts located peripherally along pleura and septal surfaces with central sparing (Figure S2C).

**P9** is an 18-year-old woman born in 2005 to consanguineous parents originating from and living in Iran (**kindred E, II.2,** Table S1). She was born after a full-term pregnancy, with no respiratory abnormalities at birth. BCG vaccination at birth resulted in disseminated BCG-osis involving the axillary, cervical and inguinal lymph nodes at the age of two months. The patient was successfully treated with a combination of isoniazid, rifampicin, ethambutol and streptomycin for two months, followed by two years of treatment with isoniazid and rifampicin. Her medical history included an episode of febrile seizure at the age of six years (normal brain MRI findings), excision surgery of an infected pilonidal cyst at the age of 11 years, multiple episodes of migraine headaches since the age of 14 years and fibrocystic disease of the breasts since the age of 14 years. The patient was hospitalized for two weeks with COVID-19 (at the age of 17 years), which was successfully treated with remdesivir and dexamethasone. During physical examination at the age of seven years, wheezing during expiration, recurrent coughs and exertional dyspnea (when climbing one flight of stairs) were noted. A chest X ray (Figure S1B) and CT scan (not shown) revealed a reticular pattern with peribronchial thickening, honeycombing and cystic lesions in the upper lobes, apical regions and subpleural regions.

#### Whole-exome and Sanger sequencing

Genomic DNA was extracted from whole blood. Whole-exome sequencing (WES) was performed with the SureSelect Human All Exon V6 (P1-P5 and P9) or the SureSelect Human All Exon 50Mb Kit (P6, P7) from Agilent. WES files for P1-P5 and P9 were filtered for homozygosity or potential compound heterozygosity for variants with a CADD score above the MSC, a GDI below 13.41 and a MAF of less than 0.01 in gnomAD. For kindred D (P6-P8), WES data for P6 and P7 were first analyzed to detect shared homozygosity or compound heterozygosity for variants with a MAF below 0.01 in the Exome Variant Server and 1000 Genome Project that were absent from our in-house controls. We also excluded variants found in dbSNP 126, 129 and 131 and those found in low-complexity regions. For confirmation of the variants and familial segregation patterns, *CCR2* exons 2 and 3 were amplified from gDNA with the following primers (5’-GGTGACAAGTGTGATCACCTGGTT-3’ and 5’-CTCCATCCACTGTCTCCCTGTAGA-3’ and 5’- TGGATCTGAGCTGGTTTGTTTTGTG-3’ and 5’- CATCTGTGAAGCCAGACGTGTGA-3’) at a Tm of 60°C, with the GoTaq DNA Polymerase (#M3005, Promega). Sanger sequencing was then performed with the BigDye Terminator v.3.1 sequencing kit (#4337455, Thermo Fisher Scientific), with analysis on an ABI Prism 3700 (Applied Biosystems).

#### Phage immunoprecipitation-sequencing

Plasma samples were collected from P1-P5 and age-matched controls. For antibody profiling by phage immunoprecipitation-sequencing (PhIP-Seq), plasma samples from patient and controls were processed as previously described^46,96^ but with the following modifications: species-specific significance cutoff values were calculated to estimate the minimum number of enriched, non-homologous peptides required to consider a sample seropositive with an in-house dataset and a generalized linear model. For each sample, we calculated virus-specific scores by dividing the counts of enriched, non-homologous peptides by the estimated score cutoff. Pooled human plasma used for IVIg (Privigen^®^ CSL Behring AG) and human IgG-depleted serum (Molecular Innovations, Inc.) were used as positive controls.

#### Generation of CCR2-deficient THP-1 cells

CCR2-deficient THP-1 cells were generated with the CRISPR/Cas9 system. The guide RNAs were designed with the Benchling design tool (https://www.benchling.com/) and inserted into lentiCRISPR v2, which was a gift from Feng Zhang (plasmid #52961; Addgene). The guide RNAs (forward: 5’-CACCGGAGATGTGGACAGCATGTTG-3’; reverse: 5’-AAACCAACATGCTGTCCA CATCTCC-3’) were designed to bind at the start of the first coding exon and to induce cutting within the 5’UTR. THP-1 cells were transduced with lentiviruses as described below and cultured for five days in the presence of puromycin before the sorting of CCR2^−^ cells. Single-cell clones were expanded in 96-well U-bottomed plates. Genomic DNA was extracted from multiple clones, amplified with the following primers: 5’-TGGCTGTTGGGTAAATCATTGATGTCTG-3’ and 5’-TAAGATGAGGACGACCAGCAT GTTGC-3’ and subjected to Sanger sequencing. The absence of CCR2 surface expression was confirmed by flow cytometry.

#### Lentivirus production and transduction

Lentiviruses for the transduction of THP-1 CCR2^KO^ cells were produced by transfecting HEK293T cells with pCMV-VSV-G (0.2 μg),^97^ pHXB2 env (0.2 μg; NIH-AIDS Reagent Program; #1069), psPAX2 (1 μg; gift from Didier Trono; Addgene plasmid #12260), pTrip-SFFV-ΔNGFR (empty vector), pTrip-SFFV-ΔNGFR-CCR2-WT or any other pTrip-SFFV-ΔNGFR containing the *CCR2* variants studied. Viral supernatants were collected 24 hours after transfection, passed through a filter with 0.45 μm pores, and protamine sulfate (8 mg/mL) was added. The viral supernatants were then added to the THP-1 cells, which were subjected to spinoculation for 2 hours at 1200 x *g* at room temperature. Transduced cells were sorted with the CD271 MicroBead Kit (#130–099-023, Miltenyi Biotec) 72 hours after transduction. The purity of the sorted cells was verified by flow cytometry with an anti-CD271 antibody (#557196, BD, 1:500).

#### Intracellular Ca^2+^ mobilization

Primary CD14^+^ monocytes from patients and healthy controls were positively selected from fresh PBMCs with anti-CD14 MicroBeads (#130–050-201, Miltenyi Biotec) used according to the manufacturer’s instructions. Intracellular Ca^2+^ mobilization was assessed as described elsewhere.^98^ In brief, 5×10^5^ THP-1 cells or primary CD14^+^ monocytes were incubated in HBSS buffer (#14025092, Thermo Fisher Scientific) supplemented with 1% FBS, 5 ng/mL Fura Red AM (#F-3021, Thermo Fisher Scientific), 2 ng/mL Fluo-4 AM (#F-14201, Thermo Fisher Scientific) and 100 mM Probenicid (#P36400, Thermo Fisher Scientific) for 30 minutes on ice. Cells were washed with HBSS buffer containing 1% FBS and incubated at 37°C before analysis on a Fortessa X20 flow cytometer. Baseline intracellular Ca^2+^ levels were determined by acquisition for 30 seconds. Cells were stimulated with 20 ng/mL CCL-2 (#300–4, Peprotech) and acquired for a total of 120 seconds. Data were analyzed with FlowJo v.10.5.3 software, based on the ratio of Fluo-4 AM to Fura Red AM. Ca^2+^ mobilization was quantified by kinetics analysis in FlowJo to determine the area under the curve (AUC) of each Ca^2+^ plot. Each plot was divided into three parts representing the baseline signal before stimulation (AUC_baseline_; 0–30 s), the gap resulting from addition of the stimulus (31 s-39 s) and the signal after stimulation (AUC_stimulation_; 40 s-120 s). The total AUC after baseline correction was calculated by subtracting the AUC_baseline_ from the AUC_stimulation_. Ca^2+^ mobilization was measured in primary CD14^+^ monocytes after stimulation with 20 ng/mL CCL-2, followed by stimulation with 1 μM ionomycin (#I0634, Merck).

#### Transwell migration assay

The migration of THP-1 cells or primary CD14^+^ monocytes was evaluated in 5 μm Transwell plates (#3421, VWR). Isolated cells were used to seed the upper compartment (2.5 × 10^6^ cells/insert) and migration medium (RPMI 1640 supplemented with 0.1% BSA (#A1595, Sigma-Aldrich)) containing 10,000 Accucount beads (#ACBP-50–10, Spherotech) with and without 20 ng/mL CCL-2 (#300–4, Peprotech) was added to the lower compartment. After 2 hours of migration at 37°C, the medium containing the cells that had migrated was removed from the lower compartment and analyzed on a Gallios flow cytometer (Beckman Coulter). The populations of cells and beads were analyzed on the basis of their size (FSC-SSC) and the number of cells that had migrated was calculated by dividing the number of cells acquired by the number of beads acquired. All data were analyzed with FlowJo v. 10.5.3 software.

#### Live-cell migration assay

Microdevices were prepared as previously described.^99^ Briefly, devices were produced with custom-made polydimethylsiloxane (PDMS) molds that had been coated with 10 μg/mL fibronectin from bovine plasma (Sigma) for 1 hour. The nuclei of the monocytes were stained for 30 minutes at 37°C, and the cells were then washed and dispensed into the wells in the devices. Medium containing 20 ng/mL of the chemokine CCL-2 was added to the wells of the devices immediately before acquisition. Migration was recorded with a Zeiss Axio Observer Z1 (Hamamatsu digital camera C11440, Carl Zeiss) microscope, with a time-lapse of 1.5 minutes and a 10X (N.A. 0.45) dry objective. Images were processed with ImageJ and single-cell trajectories were obtained with TrackMate.^100^ These trajectories were analyzed with the in-house Tracktor software developed in P.V.’s laboratory.

#### Generation of alveolar macrophage-like cells

Monocytes from patients (P1, P2, P6-P8) or healthy, unrelated controls were differentiated towards alveolar macrophage-like cells (AMLs) as previously described.^60^ Fresh peripheral monocytes were isolated from PBCMs with the classical monocyte isolation kit (#130–117-337, Miltenyi Biotec) and cultured in RPMI supplemented with 10% heat-inactivated human serum (#H4522, Merck), 80 μg/mL curosurf (Chiesi), 10 ng/mL GM-CSF, 5 ng/mL TGF-β, and 5 ng/mL IL-10 for 6 days. The cytokines and surfactant were replenished every other day. Healthy control AMLs were generated in the presence or absence of 1 μM CCR2 antagonist (#227016, Merck) or the same volume of DMSO.

#### ROS production assay

ROS production by monocytes was assessed as previously described.^101^ Whole blood was subjected to red blood cell lysis and was then incubated with dihydrorhodamine 123 (DHR) in the presence or absence of phorbol 12-myristate 13-acetate (PMA, Sigma, 400 ng/mL) for 20 minutes at 37°C. Monocytes were labeled with an anti-CD14 antibody (#558121, BD, 1:50) and samples were analyzed on a Fortessa X20 flow cytometer. Superoxide production by AMLs was assessed with a superoxide anion assay kit (#CS1000, Merck) according to the manufacturer’s instructions. Briefly, 3 × 10^4^ AMLs were plated in 96-well plates 24 hours before the experiment. Luminescence, indicating superoxide production, was recorded with a Victor Nivo plate reader (PerkinElmer) immediately after stimulation with PMA (400 ng/mL) in the presence or absence of superoxide dismutase. Data were normalized against the values for superoxide dismutase-treated wells and are expressed in luminescence units.

#### Phagocytosis of *S. aureus* bioparticles

The phagocytic capacity of isolated CD14^+^ monocytes and AMLs was evaluated with pHrodo *S. aureus* bioparticles according to the manufacturer’s instructions (#A10010, Thermo Fisher Scientific). In brief, 1×10^5^ monocytes in live imaging solution (#A14291DJ, Thermo Fisher Scientific) were dispensed into a 96-well plate and incubated with 1 mg/mL *S. aureus* bioparticles for 2 hours at 37°C. Cells were washed with live imaging solution and acquired on a Fortessa X20 flow cytometer.

#### Flow cytometry

THP-1 cells were incubated with an anti-CCR2 PE antibody (#FAB151P, R&D Systems, 1:50) or the corresponding isotype (#IC0041P, R&D Systems) and the Aqua Live/Dead Cell Stain Kit (Thermo Fisher Scientific) for 30 minutes at 4°C. Cells were washed and analyzed on a Gallios flow cytometer (Beckman Coulter). ERK phosphorylation was assessed in PBMCs stimulated with 100 ng/mL CCL-2 (#300–4, Peprotech). Cells were subsequently permeabilized and stained with anti-pERK AF647 (#561992, BD, 1:50). For AML surface marker expression, AMLs were stained with the following antibodies or their respective isotype controls for 30 minutes at 4°C: anti-CD64 FITC (#305006, BioLegend, 1:50), anti-CD163 PE (#12–1639-42, ThermoFisher Scientific, 1:50) and anti-CD206 BV421 (#321126, BioLegend, 1:50). For CCR2 and CD116 expression in different subsets of PBMCs, freshly isolated PBMCs were stained for 30 minutes at 4°C with the following antibodies: anti-CCR2 PE (#FAB151P, R&D Systems, 1:50), anti-CCR7 BV421 (#353208, BioLegend, 1:25), anti-CD1c APC-Cy7 (#331520, BioLegend, 1:50), anti-CD3 FITC (#555332, BD, 1:25), anti-CD3 PE-Cy7 (#557851, BD, 1:25), anti-CD3 BV786 (#563800, BD, 1:50), anti-CD4 BV711 (#563028, BD, 1:50), anti-CD4 APC (#130–091-232, Miltenyi Biotec, 1:25), anti-CD4 APC-Vio770 (#130–100-355, Miltenyi Biotec, 1:50), anti-CD8 BV650 (#563821, BD, 1:50), anti-CD14 FITC (#555397, BD, 1:25), anti-CD16 FITC (#555406, BD, 1:25), anti-CD16 PE-Cy7 (#302016, BioLegend, 1:50), anti-CD19 APC-Cy7 (#557791, BD, 1:50), anti-CD20-FITC (#345792, BD, 1:25), anti-CD25 BV421 (#562442, BD, 1:25), anti-CD27 BV421 (#2114120, Sony, 1:100), anti-CD45RA APC-H7 (#560674, BD, 1:50), anti-CD56 FITC (#345811), anti-CD56 PE-CF594 (#562289, BD, 1:100), anti-CD116 APC (#305913, BioLegend, 1:50), anti-CD123 PE-Cy7 (#306010, BioLegend, 1:50), anti-CD141 BV711 (#563155, BD, 1:25), anti-CD161 BV711 (#563865, BD, 1:25), anti-HLA-DR PacificBlue (#307633, BioLegend, 1:50), anti-FcεRIα BV605 (#334627, BioLegend, 1:25), anti-FoxP3 AF488 (#560047, BD, 1:10), anti-iNKT BV711 (#747720, BD, 1:25), anti-TCR-γδ BV421 (#331218, BioLegend, 1:25), and anti-TCR-Vα7.2 (#130–100-179, Miltenyi Biotec, 1:25). Samples were analyzed on a Fortessa X20 (BD) and data were analyzed with FlowJo v.10.5.3 software.

#### Immunostaining

Immunostaining was carried out on formalin-fixed-paraffin-embedded 5-μm sections on a Ventana BenchMark Ultra automated IHC/ISH stainer after antigen retrieval. Staining was performed with the following antibodies: anti-CCR2 (#NLS1899, Novus Biological, 1:2000), anti-SP-B (#WRAB-55522, Seven Hills Bioreagents, 1:4000), anti-proSP-C (#AB3786, EMD Millipore, 1:2000), anti-Aqua-porin 5 (#ab92320, Abcam, 1:1000), anti-CD20 (#120M85, Cell Marque, 1:400), anti-CD3 (#A0452, Dako, 1:400), anti-CD68 (#M0876, Dako, 1:200) and anti-CD163 (#CD163-L-CE, Leica Biosystems, 1:400). Images were visualized and captured with a digital camera mounted on a Nikon Eclipse 80i microscope using NIS-Elements Advanced Research Software v4.60.

#### Leukocyte immunophenotyping of by CyTOF

CyTOF was performed on whole blood (1 × 10^6^ cells per panel) from P1-P5 and healthy controls with the Maxpar Direct Immune Profiling Assay (Fluidigm) according to the manufacturer’s instructions. Cells were subjected to dead-cell staining overnight and were then frozen at −80°C. Acquisition was performed on a Helios machine (Fluidigm). All the samples were processed within 24 hours of sampling. Data were analyzed with OMIQ software and compared with previously acquired data for adult and age-matched controls.^61^

#### Detection of anti-IFN-γ autoantibodies

Recombinant *E. coli*-derived IFN-γ (#285-IF-100/CF, R&D Systems) was first biotinylated with EZ-Link Sulfo-NHS-LC-Biotin (#A39257, Thermo Fisher Scientific), according to the manufacturer’s instructions, with a biotin-to-protein molar ratio of 1:12. The detection reagent contained a secondary antibody [Alexa Fluor 647 goat anti-human IgG (#A21445, Thermo Fisher Scientific) diluted in Rexxip F (#P0004825, Gyros Protein Technologies; 1:500 dilution of the 2 mg/mL stock to yield a final concentration of 4 μg/mL). Phosphate-buffered saline with 0.01% Tween 20 (PBS-T) and Gyros wash buffer (#P0020087, Gyros Protein Technologies) were prepared according to the manufacturer’s instructions. Plasma or serum samples were then diluted 1:100 in 0.01% PBS-T and tested with the Bioaffy 1000 CD (#P0004253, Gyros Protein Technologies) and a Gyrolab xPand (#P0020520, Gyros Protein Technologies). Cleaning cycles were performed with 20% ethanol.

#### GM-CSF-induced STAT5 phosphorylation

PBMCs from healthy controls and patients were isolated from whole blood by Ficoll-Hypaque density centrifugation. Cells were plated at a density of 1 × 10^6^ cells/well (PBMCs) or 1 × 10^5^ cells/well (AMLs) in 100 μL RPMI supplemented with 10% FBS in 96-well V-bottomed plates. PBMCs were left unstimulated or were stimulated with 5 ng/mL GM-CSF or 100 ng/mL IL-3 (#130–093-908, Miltenyi Biotec) for 15 minutes at 37°C, before fixation and permeabilization. Extracellular labeling was performed with CD14-Pacific Blue (#558121, BD Biosciences, 1:50) and CD4-FITC (#347413, BD Biosciences, 1:50). Cell viability was determined with the Aqua Dead Cell Stain Kit and STAT5 phosphorylation was assessed by intracellular staining with a p-STAT5 (pY694)-PE antibody (BD Biosciences). Samples were assessed on a Gallios flow cytometer and analyzed with FlowJo v.10.6.2. We tested for neutralizing auto-antibodies against GM-CSF, by stimulating control PBMCs in the presence of 10% plasma obtained from patients or controls.

#### RT-qPCR

RNA from AMLs was extracted with the Quick RNA Microprep spin column kit (#R1050, Zymogen) and the remaining genomic DNA was removed by DNase I digestion. RNA was reverse-transcribed with the High-Capacity RNA-to-cDNA Kit (#4387406, Applied Bio-systems) according to the manufacturer’s instructions. We then performed quantitative reverse-transcription PCR (RT-qPCR) on the cDNA with TaqMan Fast Universal PCR Master Mix (2X), no AmpErase UNG (#4352042, Thermo Fisher Scientific) on a TaqMan ViiA7 system (Applied Biosystems) with the following probes (all from Thermo Fisher Scientific): *SPI1* (#Hs02786711_m1), *MRC1* (#Hs00267207_m1), *PPARG* (#Hs01115513_m1) and *GUSB* (#1702016)

#### RNA sequencing

Total RNA from CD14^+^ monocytes from patients and controls was isolated with the Quick RNA Microprep spin column kit (#R1050, Zymogen) according to the manufacturer’s instructions. It was then used for RNA-seq library preparation and deep sequencing at the Rockefeller University Genomics Core Facility. RNA sequencing was performed on an Illumina Novaseq, with a read length of 100 bp and a read depth of 40 M. All FASTQ files passed quality control and were aligned with the GRCh38 reference genome with STAR (2.6.1d). BAM files were converted to a raw count expression matrix with featurecount. Raw count data were normalized with DE-seq2. The ensemble IDs targeting multiple genes were collapsed (average), and a final data matrix gene was generated for downstream analysis. Absolute cell-type deconvolution analysis was performed with published software.^102^ The absolute cell-type enrichment scores are presented as dot plots generated with library ggplot2 (https://cran.r-project.org/web/packages/ggplot2/index.html). A heatmap representing classical monocyte transcript abundance profiles (*z*-score-scaled log_2_-normalized counts) was analyzed with ComplexHeatmap.^103^

#### Whole-blood activation ELISA for cytokines

Whole-blood samples from one healthy control, one healthy travel control, P1 and P2 were collected in heparin-containing collection tubes. Samples were diluted 1:2 in RPMI 1640 supplemented with 100 IU/mL penicillin and 100 μg/mL streptomycin (Thermo Fisher Scientific). Stimulation was performed in a 48-well plate. Briefly, 1 mL of whole-blood was used per well and per set of conditions. Samples were incubated with medium alone, with live BCG (*M. bovis*-BCG, Pasteur substrain) at a MOI of 20, or with BCG plus IL-12 (20 ng/mL; #219-IL, R&D Systems), or BCG plus IFN-γ (Imukin, Boehringer Ingelheim) for 48 hours at 37°C under an atmosphere containing 5% CO_2_. The supernatants were collected and used for ELISA. Data were compared to published findings for healthy and travel controls.^61^

#### Cytokine detection assays

Supernatants from whole-blood stimulation, monocytes and AMLs, and plasma, serum and BAL samples were assessed by ELISA to determine their IL-12p40 (#DP400, R&D Systems), IFN-γ (#DIF50, R&D Systems), CCL-2 (#438804, BioLegend), CCL-8 (#442204, BioLegend) or CCL-13 (#442004, BioLegend) content, in accordance with the kit manufacturer’s protocol. Plasma and BAL samples were also analyzed for the presence of inflammatory cytokines and chemokines with the following LEGENDplex^™^ assays (all from BioLegend): Human Inflammation Panel 1 (#740809), Human Inflammation Panel 2 (#740883), Human Proinflammatory Chemokine Panel 1 (#740985) and Human Proinflammatory Chemokine Panel 2 (#741183) according to the manufacturer’s instructions. Samples were analyzed by flow cytometry on a Gallios flow cytometer. Data analysis was performed with LEGENDplex Cloud-based Data Analysis Software (BioLegend). All plasma, serum and BAL samples were obtained from patients and controls at infection-free time points (culture and PCR negative).

#### Stimulation of PBMCs with BCG

Freshly thawed PBMCs were dispensed into a 96-well U-bottomed plate at a density of 2 × 10^5^ cells per well. Cells were incubated in the presence or absence of live BCG, at a multiplicity of infection of 1, with or without recombinant human IL-12 (500 pg/mL; #219-IL, R&D Systems) or recombinant human IL-23 (100 ng/mL, #1290-IL, R&D Systems). After 40 hours of stimulation, GolgiPlug (#555029, BD Biosciences) was added to each well to inhibit cytokine secretion for 8 eight hours. Cells were stained with the Zombie NIR Fixable Viability Kit (BioLegend; 1:2,000) for 15 minutes at room temperature and were then subjected to surface staining by incubation on ice for 30 minutes with a panel containing FcR blocking reagent (Miltenyi Biotec; 1:50), anti-CD3-Alexa Fluor 532 (#58–0038-42, eBioscience, 1:50), anti-γδTCR-FITC (#11–9959-41, eBioscience, 1:50), anti-Vδ2-APC/Fire 750 (#331419, BioLegend,; 1:100), anti-CD56-BV605 (#362537, BioLegend, 1:100), anti-CD4-BV750 (#566356, BD Biosciences, 1:800), anti-CD8a-Pacific Blue (#344717, BioLegend, 1:100), anti-Vα7.2 TCR-APC (#351708, BioLegend, 1:100), anti-Vα24-Jα18-PE/Cy7 (#342912, BioLegend, 1:100), anti-CD20-BV785 (#302356, BioLegend, 1:200) and anti-PD- 1-PE (eBioscience, #12–9969-42; 1:100 dilution) antibodies. Cells were fixed by incubation with 2% paraformaldehyde in PBS for 15 minutes on ice. Cells were then permeabilized/stained by incubation overnight at −20°C in the permeabilization buffer from the Nuclear Transcription Factor Buffer Set (BioLegend), with an intracellular cytokine panel containing FcR blocking reagent, anti-IFN-γ-BV711 (#502540; BioLegend, 1:50), anti-TNF-BV510 (#502950, BioLegend, 1:50) and anti-IL-10-PE/Dazzle594 (#506812, BioLegend, 1:50) antibodies. As a positive control, cells in a separate well were stimulated by incubation with PMA (25 ng/mL) and ionomycin (500 nM) for one hour without GolgiPlug, followed by a further 7 hours with GolgiPlug (for intracellular cytokine staining). Cells were acquired with an Aurora cytometer (Cytek). Data were manually gated with FlowJo.

#### Assessment of STAT phosphorylation by CyTOF

Fresh blood from three healthy controls, P1 and P2 was either left unstimulated, or was stimulated with IL-23 (100 ng/mL; #1290-IL, R&D Systems), IL-12 (100 ng/mL; #219-IL, R&D Systems), IFN-γ (1000 IU/mL) or IFN-α2b (10^5^ IU/mL; Intron A, Merk). After 20 minutes of incubation, samples were fixed and frozen in protein stabilizer (PROT1, Smart Tube Inc.). Stabilized blood samples were thawed according to the manufacturer’s recommended protocol and washed with barcode permeabilization buffer (Fluidigm). Samples were then uniquely barcoded with the Cell-ID 20-Plex Pd Barcoding Kit (Fluidigm), washed and pooled. Non-specific binding was prevented with Fc- and heparin-block. Cells were then incubated with an antibody cocktail for surface markers to identify major immune populations. All the antibodies were purchased pre-conjugated or were conjugated in-house with X8 MaxPar conjugation kits (Fluidigm). After surface staining, the samples were washed, permeabilized in methanol, stored at −80°C overnight, washed again, blocked with heparin and stained with a cocktail of antibodies against intracellular targets. Cells were washed again and incubated in freshly diluted 2.4% formaldehyde containing 125 nM Ir Intercalator (Fluidigm), 0.02% saponin and 30 nM OsO_4_ (ACROS Organics) for 30 minutes at room temperature. For acquisition, samples were washed with PBS + 0.2% BSA, PBS, and CAS buffer (Fluidigm). The final suspension in CAS buffer contained 10^6^ cells per mL and a 1/20 dilution of EQ beads (Fluidigm). Following routine instrument optimization, samples were acquired at a rate of < 300 events per second on a Helios mass cytometer (Fluidigm) with a modified wide-bore injector (Fluidigm). FCS files were normalized and concatenated with Fluidigm acquisition software and deconvoluted with a MATLAB-based debarcoding application. The resulting files were analyzed with OMIQ as previously described.^93^

#### ScRNA-seq of cryopreserved BAL cells

Cryopreserved BAL cells from two healthy adult controls and two CCR2-deficient patients (P1 and P2) were analyzed. Cells were filtered with a 70 μm MACS SmartStrainer (#130–098-462, Miltenyi) to remove large debris, washed three times with PBS plus 0.5% FBS, and finally filtered with a 40 μm Falcon Cell Strainer (#352340, Corning). Single cells were then captured with the 10X Genomics Chromium chip. Libraries were prepared with Chromium Single Cell 3’ Reagent Kit (v3 Chemistry) and sequenced with an Illumina NovaSeq 6000 sequencer. Sequence data were preprocessed with CellRanger. Depending on the number of cells available, approximately 4,000 to 15,000 cells were captured per sample, with a mean of 15,000 to 75,000 reads per cell. Data were filtered manually according to common quality-control metrics (i.e., the number of unique genes detected in each cell, the total number of RNA molecules detected in each cell, and the percentage of reads that mapping to the mitochondrial genome) with Seurat.^104^

We performed cell-type label transfer to identify alveolar macrophage-like cells. Briefly, previously published BAL cell datasets for three healthy donors (C51, C52, and C100)^105^ [GSE145926] were used as a reference for the FindTransferAnchors function implemented in Seurat. Cells labeled as macrophages in the original datasets but not assigned to any of the four macrophage clusters (Groups 1 ~4) were not used for label transfer. The Group 4 macrophages were used as a proxy for alveolar macrophages, as described in the original study.^105^ In addition to label transfer analysis, we also conducted an unsupervised clustering analysis with the FindNeighbors and FindClusters functions in Seurat. Clusters were annotated by manual inspection, with the aid of predictions based on the label transfer data. Differential expression analysis was performed with the FindMarkers function in Seurat. Genes with a mean log_2_ fold-change >=0.5 or <= −0.5 and an FDR-adjusted P value < 0.05 were considered to be differentially expressed. All analyses were performed in R v4 (http://www.R-project.org/).

#### ScRNA-seq of differentiated iPSCs

We used a modified version of the previously described EB-based hematopoietic differentiation protocol^92,106^ to differentiate human induced pluripotent stem cells (iPSCs) into hematopoietic cells. To initiate differentiation, we generated embryoid bodies (EBs) from detached iPSC colonies (#MHHi015-A, hPSCreg) using 2 mg/mL collagenase IV (#17104019, Invitrogen) and cultivated them on an orbital shaker in iPSC-medium without bFGF but supplemented with 10 μM Rock inhibitor (Y-27632; Tocris). After 2–3 days, the medium was changed, and after 5 days, mature EBs were manually selected and transferred to adherent plates in differentiation medium I supplemented with either 50 ng/ml IL-3 or a combination of 25 ng/ml IL-3 (Peprotech) and 50 ng/ml M-CSF (Peprotech). We acquired samples for single-cell RNA transcriptome analysis at the mature EB stage and also 8 or 16 days after initiation of hematopoietic differentiation medium I for both cytokine conditions. EB and adherent myeloid-cell-forming-complexes were dissociated with TrypLE Express (#12605028, Thermo Fischer Scientific) for 10–30 minutes at 37°C and filtered through 70 μm mesh. Subsequently, the single-cell suspension was stained for CD34 expression (#11–0349, 1:50, eBioscience) and with DAPI (Sigma) and was further enriched by sorting (FACS Aria, BD) for live and CD34^+^ cells.

#### Library preparation and sequencing

Library preparation for single-cell mRNA-Seq analysis was performed according to the Chromium NextGEM Single Cell 3ʹ Reagent Kits v3.1 User Guide (Manual Part Number CG000204 Rev B; 10x Genomics). A twofold excess of cells was loaded to the 10x controller in the specified volume in order to reach a target number of 5000 cells per sample. Fragment length distribution of generated cDNAs and libraries was monitored using ‘Bioanalyzer High Sensitivity DNA Assay’ (5067–4626; Agilent Technologies). Quantification of libraries was performed by use of the ‘Qubit dsDNA HS Assay Kit’ (Q32854; ThermoFisher Scientific).

Equal molar proportions of two (N2140-N2141) or five (N2142-N2146) generated libraries were pooled accordingly, denatured with NaOH, and were finally diluted to 2 pM according to the ‘Denature and Dilute Libraries Guide’ (Document # 15048776 v02; Illumina). 1.3 ml of the denatured pool was sequenced on an Illumina NextSeq 550 sequencer using one High Output Flowcell for 75 cycles and 400 million clusters (#20024906; Illumina) each, for both subseries (N2140-N2141) and (N2142-N2146). Sequencing was performed according to the following settings: 28bp as sequence read 1; 56bp as sequence read 2; 8bp as index read 1; no index read 2.

#### Raw data processing

The proprietary 10x Genomics CellRanger pipeline (v4.0.0) was used with default parameters except for the expect-cells setting. Initially, the mkfastq command was used to demultiplex the data from the raw BCL sequencer images, given the supplied sample sheet with the utilized 10x barcodes. This step resulted in standard FASTQ files and 10x specific metadata. Next, CellRanger was used to align read data to the reference genome provided by 10x Genomics (Human reference dataset refdata-gex-GRCh38–2020-A) using STAR aligner. Aligned reads were counted per gene and clustering and summary statistics were calculated accordingly.

#### ScRNA-seq computational analysis

To process the unique molecular identifier count matrix and normalize data for all samples, Seurat (version 4.3.0)^104^ R package was utilized. Filtering criteria were applied using Seurat, wherein cells were excluded based on the number of genes (<200), gene count (<1000), and percentage of mitochondrial RNA (>16%). Cells identified as doublets using DoubletFinder^107^ (pN = 0.25, pK = 0.01) were also removed. After merging all datasets, UMI counts, mitochondrial RNA, and cell cycle variables were scaled by regressing. The RunPCA function was used for performing Principal Component Analysis (PCA) based on the 3,000 genes. After PCA dimensionality reduction and Louvain clustering, the datasets were further analyzed. To annotate the data set, a logistic regression (LR) model^108^ was built based on publicly available single-cell transcriptomic data sets.^58^ Mean prediction probability was calculated per Louvain cell cluster, and each cluster was labelled based on the LR model with the highest mean prediction. For downstream analysis, the data were subset to hematopoietic and hemogenic endothelial clusters.

### QUANTIFICATION AND STATISTICAL ANALYSIS

Statistical analyses were performed with GraphPad Prism 9.3.1. Mann-Whitney, ANOVA or Kruskal-Wallis tests were performed. In the relevant figures, n.s. indicates not significant, ***P ≤ 0.001; and *P ≤ 0.05.

## Supplementary Material

MMC3

MMC2

MMC1

1

## Figures and Tables

**Table:** KEY RESOURCES TABLE

REAGENT or RESOURCE	SOURCE	IDENTIFIER
Antibodies		

Human CD34-FITC	eBioscience	Cat# 11-0349; RRID:AB_1518732
Human CD3-Alexa Fluor 532	eBioscience	Cat# 58-0038-42; RRID:AB_11218675
Human γδTCR-FITC	eBioscience	Cat# 11-9959-41; RRID:AB_10669048
Human Vδ2-APC/Fire 750	BioLegend	Cat# 331419; RRID:AB_2687325
Human CD56-BV605	BioLegend	Cat# 362537; RRID:AB_2565855
Human CD4-BV750	BD Biosciences	Cat# 566356; RRID:AB_2662287
Human CD8a-Pacific Blue	BioLegend	Cat# 344717; RRID:AB_10551616
Human Vα7.2 TCR-APC	BioLegend	Cat# 351708; RRID:AB_10933246
Human Vα24-Jα18-PE/Cy7	BioLegend	Cat# 342912; RRID:AB_2562230
Human CD20-BV785	BioLegend	Cat# 302356; RRID:AB_2566316
Human PD-1-PE	eBioscience	Cat# 12-9969-42; RRID:AB_10736473
Human IFN-γ-BV711	BioLegend	Cat# 502540; RRID:AB_2563506
Human TNF-BV510	BioLegend	Cat# 502950; RRID:AB_2565860
Human IL-10-PE/Dazzle594	BioLegend	Cat# 506812; RRID:AB_2632783
Human CD14-Pacific Blue	BD Biosciences	Cat# 558121; RRID:AB_397041
Human CD4-FITC	BD Biosciences	Cat# 347413; RRID:AB_400297
Human p-STAT5 (pY694)-PE	BD Biosciences	Cat# 612567; RRID:AB_399858
Alexa Fluor 647 goat anti-human IgG	Thermo Fisher Scientific	Cat# A21445; RRID:AB_2535862
Human CD271-PE	BD Biosciences	Cat# 557196; RRID:AB_396599
Human CCR2-PE	R&D Systems	Cat# FAB151P; RRID:AB_2106270
Mouse IG2b isotype control-PE	R&D Systems	Cat# IC0041P; RRID:AB_357249
Human pERK-AF647	BD Biosciences	Cat# 561992; RRID:AB_10896138
Human CCR7-BV421	BioLegend	Cat# 353208; RRID:AB_11203894
Human CD1c-APC-Cy7	BioLegend	Cat# 331520; RRID:AB_10644008
Human CD3-FITC	BD Biosciences	Cat# 555332; RRID:AB_395739
Human CD3-PE-Cy7	BD Biosciences	Cat# 557851; RRID:AB_396896
Human CD3-BV786	BD Biosciences	Cat# 563800; RRID:AB_2738487
Human CD4-BV711	BD Biosciences	Cat# 563028; RRID:AB_2737961
Human CD4-APC	Miltenyi	Cat# 130-091-232; RRID:AB_871690
Human CD4-APC-Vio770	Miltenyi	Cat# 130-100-355; RRID:AB_2657995
Human CD8-BV650	BD Biosciences	Cat# 563821; RRID:AB_2744462
Human CD14-FITC	BD Biosciences	Cat# 555397; RRID:AB_395798
Human CD16-FITC	BD Biosciences	Cat# 555406; RRID:AB_395806
Human CD16-PE-Cy7	BioLegend	Cat# 302016; RRID:AB_314216
Human CD19-APC-Cy7	BD Biosciences	Cat# 557791; RRID:AB_396873
Human CD20-FITC	BD Biosciences	Cat# 345792; RRID:AB_2868818
Human CD25-BV421	BD Biosciences	Cat# 562442; RRID:AB_11154578
Human CD27-BV421	Sony	Cat# 2114120; RRID:AB_2861442
Human CD45RA-APC-H7	BD Biosciences	Cat# 560674; RRID:AB_1727497
Human CD56-FITC	BD Biosciences	Cat# 345811; RRID:AB_2868832
Human CD56-PE-CF594	BioLegend	Cat# 562289; RRID:AB_11152080
Human CD116-APC	BioLegend	Cat# 305913; RRID:AB_2860814
Human CD123-PE-Cy7	BioLegend	Cat# 306010; RRID:AB_493576
Human CD141-BV711	BD Biosciences	Cat# 563155; RRID:AB_2738033
Human CD161-BV711	BD Biosciences	Cat# 563865; RRID:AB_2738457
Human HLA-DR-PacificBlue	BioLegend	Cat# 307633; RRID:AB_1595444
Human FcεRIα-BV605	BioLegend	Cat# 334627; RRID:AB_2566505
Human FoxP3-AF488	BD Biosciences	Cat# 560047; RRID:AB_1645349
Human iNKT-BV711	BD Biosciences	Cat# 747720; RRID:AB_2872199
Human TCR-γδ-BV421	BioLegend	Cat# 331218; RRID:AB_2562317
Human TCR-Vα7.2	Miltenyi	Cat# 130-100-179; RRID:AB_2653673
Human CD64-FITC	BioLegend	Cat# 305006; RRID:AB_314490
Mouse IgG1 κ Isotype control-FITC	BioLegend	Cat# 400108; RRID:AB_396090
Human CD163-PE	eBioscience	Cat# 12-1639-42; RRID:AB_1963570
Mouse IgG1 κ Isotype control-PE	eBioscience	Cat# 12-4714-82; RRID:AB_470060
Human CD206-BV421	BioLegend	Cat# 321126; RRID:AB_2563839
Mouse IgG1 κ Isotype control-BV421	BioLegend	Cat# 400158; RRID:AB_11150232
Human CCR2	Novus Biologicals	Cat# NLS1899; RRID: AB_10003193
Human SP-B	Seven Hills Bioreagents	Cat# WRAB-55522; RRID: AB_2938816
Human proSP-C	EMD Millipore	Cat# AB3786; RRID: AB_91588
Human Aquaporin 5	Abcam	Cat# ab92320; RRID: AB_2049171
Human CD20 (L26)	Cell Marque	Cat# 120M85; RRID: AB_1158146
Human CD3	Dako	Cat# A0452; RRID: AB_2335677
Human CD68	Dako	Cat# M0876; RRID: AB_2074844
Human CD163	Leica Biosystems	Cat# CD163-L-CE; RRID: AB_2920861

Bacterial and virus strains		

Mycobacterium bovis BCG	Vogt and Nathan, 2011, PMID: 21911939	N/A

Biological samples		

Peripheral blood mononuclear cells from indicated individuals	This manuscript	N/A
Plasma from indicated individuals	This manuscript	N/A
Serum from indicated individuals	This manuscript	N/A
Biopsies from indicated individuals	This manuscript	N/A
Bronchoalveolar alveolar lavage from indicated individuals	This manuscript	N/A

Chemicals, peptides, and recombinant proteins		

Recombinant IFN-γ−1b (Imukin)	Clinigen Healthcare France	Cat# 3400955776789
Recombinant IFN-γ	R&D Systems	Cat# 285-IF-100/CF
Recombinant interferon alpha-2b (Introna)	MSD France	Cat# 3400934956287
DpnI	New England Biolabs	Cat# R0176L
Aqua Dead Cell Stain kit	Aqua Dead Cell Stain kit	Cat# L34957
Recombinant human Interleukin-12	R&D Systems	Cat# 219-IL-025
Recombinant human Interleukin-23	R&D Systems	Cat# 1290-IL-010
Recombinant human GM-CSF	R&D Systems	Cat# 215-GM
Protamine sulfate	Merck	Cat# P3369-10G
Recombinant human CCL-2	Peprotech	Cat# 300-4
Recombinant human CXCL-12	Peprotech	Cat# 300-28A
Recombinant human Interleukin-3	Miltenyi Biotec	Cat# 130-093-908
Recombinant human Interleukin-3	Peprotech	Cat# 200-03
Recombinant human M-CSF	Peprotech	Cat# 300-25
GolgiPlug	BD Biosciences	Cat# 555029
Zombie NIR^™^ Fixable Viability Kit	BioLegend	Cat# 423105
Lymphoprep	StemCell	Cat# 07801
FcBlock	Miltenyi Biotec	Cat# 130-059-901
Ionomycin calcium salt	Merck	Cat# I0634
Phorbol 12-myristate 13-acetate	Merck	Cat#
Collagenase IV	Thermo Fisher Scientific	Cat# 17104019
Rock inhibitor	Tocris	Cat# Y-27632
TrypLE Express	Thermo Fisher Scientific	Cat# 12605028
pHrodo *S. aureus* bioparticles	Thermo Fisher Scientific	Cat# A10010
Dihydrorhodamine 123	Merck	Cat# D1054-2MG
Fluo-4 AM	Thermo Fisher Scientific	Cat# F-14201
Fura Red AM	Thermo Fisher Scientific	Cat# F-3021
Probenicid	Thermo Fisher Scientific	Cat# P36400
HBSS buffer	Thermo Fisher Scientific	Cat# 14025092
GoTaq DNA Polymerase	Promega	Cat# M3005
Fibronectin bovine plasma	Merck	Cat# F1141-5MG
Recombinant human GM-CSF	BioLegend	Cat#572914
Recombinant human TGF-β	BioLegend	Cat#781804
Recombinant human IL-10	BD Biosciences	Cat#554611
Curosurf	Chiesi	N/A
Live imaging solution	Thermo Fisher Scientific	Cat# A14291DJ

Critical commercial assays		

Quick-RNA Micro-Prep Kit	Zymo	Cat# R1051
ELISA IL-12p40	R&D Systems	Cat# DP400
ELISA IFN-γ	R&D Systems	Cat# DIF50
ELISA CCL-2	BioLegend	Cat# 438804
CD14 MicroBeads	Miltenyi	Cat# 130-050-201
Classical monocyte isolation kit	Miltenyi	Cat# 130-117-337
CD271 MicroBeads	Miltenyi	Cat# 130-099-023
Big Dye Terminator v3.1 cycle sequencing kit	Applied Biosystems	Cat# 4337455
SureSelect Human All Exon V6	Agilent	Cat# 5190-8864
SureSelect Human All Exon 50Mb	Agilent	Cat# 5190-4628
Bioaffy 1000 CD	Gyros Protein Technologies	Cat# P0004253
Gyrolab xPand	Gyros Protein Technologies	Cat# P0020520
LEGENDplex^™^ Human Inflammation Panel 1	BioLegend	Cat# 740809
LEGENDplex^™^ Human Inflammation Panel 2	BioLegend	Cat# 740883
LEGENDplex^™^ Human Proinflammatory Chemokine Panel 1	BioLegend	Cat# 740985
LEGENDplex^™^ Human Proinflammatory Chemokine Panel 2	BioLegend	Cat# 741183
High-Capacity RNA-to-cDNA Kit	Applied Biosystems	Cat# 4387406
Superoxide Anion Assay Kit	Merck	Cat# CS1000
TaqMan Fast Universal PCR Master Mix (2X), no AmpErase UNG	Thermo Fisher Scientific	Cat# 4352042

Deposited data		

scRNA-seq on iPSC-derived hematopoietic cells	This manuscript	Gene expression omnibus (GSE242972)
RNA-seq on primary monocytes	This manuscript	Sequence Read Archive (PRJNA949597)
scRNA-seq on cryopreserved BAL samples	This manuscript	Sequence Read Archive (PRJNA949597)

Experimental models: Cell lines		

THP1 WT	ATCC	Cat# TIB-202, RRID:CVCL_0006
THP1 CCR2^KO^	This manuscript	N/A
iPSC healthy control clone 16	Lachmann et al.^92^, PMID: 24279725	#MHHi015-A

Oligonucleotides		

sgRNA CCR2 F 5’-CACCGGAGATGTGGACAGCATGTTG-3’	Eurofins	N/A
sgRNA CCR2 R 5’-AAACCAACATGCTGTCCACATCTCC-3’	Eurofins	N/A
gDNA *CCR2* F 5’-TGGCTGTTGGGTAAATCATTGATGTCTG-3’	Eurofins	N/A
gDNA *CCR2* R 5’-TAAGATGAGGACGACCAGCATGTTGC-3’	Eurofins	N/A
gDNA *CCR2* Exon 2 F 5’-GGTGACAAGTGTGATCACCTGGTT-3’	Eurofins	N/A
gDNA *CCR2* Exon 2 R 5’-CTCCATCCACTGTCTCCCTGTAGA-3’	Eurofins	N/A
gDNA *CCR2* Exon 3 F 5’-TGGATCTGAGCTGGTTTGTTTTGTG-3’	Eurofins	N/A
gDNA *CCR2* Exon 3 R 5’-CATCTGTGAAGCCAGACGTGTGA-3’	Eurofins	N/A
*CCR2* pTRIP subcloning F 5’- ACCTGGATCCACTAGTATGCTGTCCACATCTCGTTC-3’	Eurofins	N/A
*CCR2B* pTRIP subcloning R 5’- AGGTCTCGAGGTCGACTTATAAACCAGCCGAGACTTCC-3’	Eurofins	N/A
*CCR2A* pTRIP subcloning R 5’- AGGTCTCGAGGTCGACTTAGGCTCCTTCTTTGTCCTGAA-3’	Eurofins	N/A
*CCR2A/B* p.T21Pfs*18 F 5’-GCGGTGAAGAAGTACCACCACCTTTTTT-3’	Eurofins	N/A
*CCR2A/B* p.T21Pfs*18 R 5’-AAAAAAGGTGGTGGTACTTCTTCACCGC-3’	Eurofins	N/A
*CCR2A/B* p.M61R F 5’-TTGTGGGCAACAGGCTGGTCGTCCTC-3’	Eurofins	N/A
*CCR2A/B* p.M61R R 5’-GAGGACGACCAGCCTGTTGCCCACAA-3’	Eurofins	N/A
*CCR2A/B* p.V64I F 5’-CATGCTGGTCATCCTCATCTT-3’	Eurofins	N/A
*CCR2A/B* p.V64I R 5’-AAGATGAGGATGACCAGCATG-3’	Eurofins	N/A
*CCR2A/B* p.L119R F 5’-TTATTCACAGGGCGGTATCACATCGG-3’	Eurofins	N/A
*CCR2A/B* p.L119R R 5’-CCGATGTGATACCGCCCTGTGAATAA-3’	Eurofins	N/A
*CCR2A/B* p.L133F F 5’-CTTCATCATCTTCCTGACAAT-3’	Eurofins	N/A
*CCR2A/B* p.L133F R 5’-ATTGTCAGGAAGATGATGAAG-3’	Eurofins	N/A
*CCR2A/B* p.P214_L215del R 5’-CAGGACCAGCCCCAAAATGT-3’	Eurofins	N/A
*CCR2A/B* p.P214_L215del F 5’-CTCATCATGGTCATCTGCTACTCGG-3’	Eurofins	N/A
*CCR2A/B* p.K227_R243del F 5’-GTCATCTTCACCATCATGATTGTTTACT-3’	Eurofins	N/A
*CCR2A/B* p.K227_R243del R 5’-CAGGATTCCCGAGTAGCAGAT-3’	Eurofins	N/A
*CCR2A/B* p.T296N F 5’-CTCTTGGGATGAATCACTGCTGCAT-3’	Eurofins	N/A
*CCR2A/B* p.T296N R 5’-ATGCAGCAGTGATTCATCCCAAGAG-3’	Eurofins	N/A
*CCR2B* p.R327C F 5’-ACATCACCAAGTGCTTCTGCAAAC-3’	Eurofins	N/A
CCR2B p.R327C R 5’-GTTTGCAGAAGCACTTGGTGATGT-3’	Eurofins	N/A
CCR2A p.F316L F 5’-GTTCAGAAGCCTTTTACACATAGCTCTTGGC-3’	Eurofins	N/A
CCR2A p.F316L R 5’-GCCAAGAGCTATGTGTAAAAGGCTTCTGAAC-3’	Eurofins	N/A
CCR2A p.G355D F 5’-GATGGTCGTGAAAAAGGAAAG-3’	Eurofins	N/A
CCR2A p.G355D R 5’-CTTTCCTTTTTCACGACCATC-3’	Eurofins	N/A
*GUSB*	Thermo Fisher Scientific	Cat# 1702016
*SPI1*	Thermo Fisher Scientific	Cat# Hs02786711_m1
*MRC1*	Thermo Fisher Scientific	Cat# Hs00267207_m1
*PPARG*	Thermo Fisher Scientific	Cat# Hs01115513_m1

Recombinant DNA		

plentiCRISPR v2	Addgene	Cat# 52961
psPAX2	Addgene	Cat# 12260
pCMV-VSV-G	Addgene	Cat# 8454
pHXB2-Env	NIH-AIDS Reagent Program	Cat# 1069
pTrip-SFFV-ΔNGFR-2A	Philippot et al.,^93^ PMID: 36763636	N/A
pTrip-SFFV-CCR2A-ΔNGFR-2A	This manuscript	N/A
pTrip-SFFV-CCR2A-p.T21Pfs*18-ΔNGFR-2A	This manuscript	N/A
pTrip-SFFV-CCR2A-p.M61R-ΔNGFR-2A	This manuscript	N/A
pTrip-SFFV-CCR2A-p.V64I-ΔNGFR-2A	This manuscript	N/A
pTrip-SFFV-CCR2A-p.L119R-ΔNGFR-2A	This manuscript	N/A
pTrip-SFFV-CCR2A-p.L133F-ΔNGFR-2A	This manuscript	N/A
pTrip-SFFV-CCR2A-p.P214_L215del-ΔNGFR-2A	This manuscript	N/A
pTrip-SFFV-CCR2A-p.T296N-ΔNGFR-2A	This manuscript	N/A
pTrip-SFFV-CCR2A-p.F316L-ΔNGFR-2A	This manuscript	N/A
pTrip-SFFV-CCR2A-p.G355D-ΔNGFR-2A	This manuscript	N/A
pTrip-SFFV-CCR2A-p.K227_R243del-ΔNGFR-2A	This manuscript	N/A
pTrip-SFFV-CCR2B-ΔNGFR-2A	This manuscript	N/A
pTrip-SFFV-CCR2B-p.T21Pfs*18-ΔNGFR-2A	This manuscript	N/A
pTrip-SFFV-CCR2B-p.M61R-ΔNGFR-2A	This manuscript	N/A
pTrip-SFFV-CCR2B-p.V64I-ΔNGFR-2A	This manuscript	N/A
pTrip-SFFV-CCR2B-p.L119R-ΔNGFR-2A	This manuscript	N/A
pTrip-SFFV-CCR2B-p.L133F-ΔNGFR-2A	This manuscript	N/A
pTrip-SFFV-CCR2B-p.P214_L215del-ΔNGFR-2A	This manuscript	N/A
pTrip-SFFV-CCR2B-p.T296N-ΔNGFR-2A	This manuscript	N/A
pTrip-SFFV-CCR2B-p.R327C-ΔNGFR-2A	This manuscript	N/A
pTrip-SFFV-CCR2B-p.K227_R243del-ΔNGFR-2A	This manuscript	N/A

Software and algorithms		

R	The R Project for Statistical Computing	https://www.r-project.org
Uni-form Mani-fold Approximation and Projection (UMAP)	Becht et al.^94^ (PMID 30531897)	v.0.3.5
STAR (2.6.1d)	Dobin et al.^95^ PMID: 23104886	https://github.com/alexdobin/STAR
Cell Ranger	10X Genomics	v3.0.1

Other		

6.5 mm Transwell^®^ with 5.0 μm Pore Polycarbonate Membrane Insert	Corning	Cat# 3421
AccuCount Blank Particles	Spherotech	Cat# ACBP-50-10

## Highlights

- Inherited human CCR2 deficiency impairs pulmonary recruitment of monocytes
- Insufficient attraction of monocytes reduces the counts of alveolar macrophages
- Reduced counts of human alveolar macrophages underlie chronic lung disease
- Inherited human CCR2 deficiency also impairs protective immunity to mycobacteria
