# Human inherited CCR2 deficiency underlies progressive polycystic lung disease

**DOI:** 10.1016/j.cell.2024.05.021

**Published:** 2024-05-21

**Authors:** Anna-Lena Neehus, Brenna Carey, Marija Landekic, Patricia Panikulam, Gail Deutsch, Masato Ogishi, Carlos A. Arango-Franco, Quentin Philippot, Mohammadreza Modaresi, Iraj Mohammadzadeh, Melissa Corcini Berndt, Darawan Rinchai, Tom Le Voyer, Jérémie Rosain, Mana Momenilandi, Marta Martin-Fernandez, Taushif Khan, Jonathan Bohlen, Ji Eun Han, Alexandre Deslys, Mathilde Bernard, Tania Gajardo-Carrasco, Camille Soudée, Corentin Le Floc’h, Mélanie Migaud, Yoann Seeleuthner, Mi-Sun Jang, Eirini Nikolouli, Simin Seyedpour, Hugues Begueret, Jean-François Emile, Pierre Le Guen, Guido Tavazzi, Costanza Natalia Julia Colombo, Federico Capra Marzani, Micol Angelini, Francesca Trespidi, Stefano Ghirardello, Nasrin Alipour, Anne Molitor, Raphael Carapito, Mohsen Mazloomrezaei, Hassan Rokni-Zadeh, Majid Changi-Ashtiani, Chantal Brouzes, Pablo Vargas, Alessandro Borghesi, Nico Lachmann, Seiamak Bahram, Bruno Crestani, Michael Fayon, François Galode, Susanta Pahari, Larry S. Schlesinger, Nico Marr, Dusan Bogunovic, Stéphanie Boisson-Dupuis, Vivien Béziat, Laurent Abel, Raphael Borie, Lisa R. Young, Robin Deterding, Mohammad Shahrooei, Nima Rezaei, Nima Parvaneh, Daniel Craven, Philippe Gros, Danielle Malo, Fernando E. Sepulveda, Lawrence M. Nogee, Nathalie Aladjidi, Bruce C. Trapnell, Jean-Laurent Casanova, Jacinta Bustamante

(Cell *187*, 390–408.e1–e14; January 18, 2024)

Our paper reported that the chemokine receptor CCR2 is important for recruitment and maturation of human macrophages in the lung. In the original version of the paper, Michael Fayon and François Galode, who contributed the description of clinical diseases in two patients (P1–P2) and provided resource materials, were inadvertently left off the author list. To correct this oversight, we now include them as authors on the paper with their respective affiliations. All co-authors on the manuscript have approved this addition. The spelling of Dusan Bogunovic’s surname has also been corrected in the updated version. The paper has now been corrected online. We apologize for any inconvenience.

