# Supplementary material for: Human inherited CCR2 deficiency underlies progressive polycystic lung disease
Source: Cell. Author manuscript; Available in PMC 2024 Feb 5. (PMC10842692; doi:10.1016/j.cell.2023.11.036)
Supplement: MMC1 [file NIHMS1949155-supplement-MMC1.pdf]

Cell, Volume *187*

Supplemental information

Human inherited CCR2 deficiency underlies progressive polycystic lung disease

Anna-Lena Neehus, Brenna Carey, Marija Landekic, Patricia Panikulam, Gail Deutsch, Masato Ogishi, Carlos A. Arango-Franco, Quentin Philippot, Mohammadreza Modaresi, Iraj Mohammadzadeh, Melissa Corcini Berndt, Darawan Rinchai, Tom Le Voyer, Jérémie Rosain, Mana Momenilandi, Marta Martin-Fernandez, Taushif Khan, Jonathan Bohlen, Ji Eun Han, Alexandre Deslys, Mathilde Bernard, Tania Gajardo-Carrasco, Camille Soudée, Corentin Le Floc'h, Mélanie Migaud, Yoann Seeleuthner, Mi-Sun Jang, Eirini Nikolouli, Simin Seyedpour, Hugues Begueret, Jean-François Emile, Pierre Le Guen, Guido Tavazzi, Costanza Natalia Julia Colombo, Federico Capra Marzani, Micol Angelini, Francesca Trespidi, Stefano Ghirardello, Nasrin Alipour, Anne Molitor, Raphael Carapito, Mohsen Mazloomrezaei, Hassan Rokni-Zadeh, Majid Changi-Ashtiani, Chantal Brouzes, Pablo Vargas, Alessandro Borghesi, Nico Lachmann, Seiamak Bahram, Bruno Crestani, Susanta Pahari, Larry S. Schlesinger, Nico Marr, Dusan Bugonovic, Stéphanie Boisson-Dupuis, Vivien Béziat, Laurent Abel, Raphael Borie, Lisa R. Young, Robin Deterding, Mohammad Shahrooei, Nima Rezaei, Nima Parvaneh, Daniel Craven, Philippe Gros, Danielle Malo, Fernando E. Sepulveda, Lawrence M. Nogee, Nathalie Aladjidi, Bruce C. Trapnell, Jean-Laurent Casanova, and Jacinta Bustamante

Table S1. Demographics, clinical characteristics, diagnostic tests, natural course of disease, and current status of the patients with CCR2 deficiency, related to Figure 1.

Kindred	A		B	C		D			E
Patient	1	2	3	4	5	6	7	8	9
Sex	Female	Female	Female	Male	Male	Female	Female	Female	Female
Place of birth (parental origin)	France (Algeria)	France (Algeria)	Iran	Iran	Iran	USA	USA	USA	Iran
Year of birth	2010	2014	2010	2010	2012	2001	2006	2008	2005
Consanguinity	Yes	Yes	Yes	Yes	Yes	No	No	No	Yes
Birth history/complications	Term/none	Term/none	Term/none	Term/none	Term/none	Term/none	Term/none	Term/none	Term/none
BCG vaccination in infancy, Response	Yes, Lymphadenitis (BCG-itis)	Yes, No adverse events	Yes, Lymphadenitis (BCG-itis)	Yes, No adverse events	Yes, No adverse events	No, Not applicable	No, Not applicable	No, Not applicable	Yes, dissaminated BCG disease
Clinical presentation									
Digital clubbing	?	?	?	Yes	Yes	Yes	Yes	No	No
Growth pattern	Normal	Growth failure	Normal	?	?	Growth failure	Growth failure	Normal	Normal
Age at onset of symptoms, years	4	4	3	11	9	7	2.4	(19 months)	2 months
Symptoms and signs	DOE, pneumonia	Cough, DOE	NA	Cough, DOE	Cough, DOE	DOE	Cough, DOE	None	Cough, DOE
Age at evaluation (this study), years	12	8	12	12	10	21	16	14	18
Diagnostic testing, results									
SpO_2, % (FiO_2)	?	97	?	96	?	97 (room air)	98 (room air)	97 (room air)	96 (room air)
VO_2 max, % predicted	47	ND	?	?	?	?	?	?	ND
Sweat chloride test	ND	ND	?	Yes, negative	Yes, negative	Yes, negative	Yes, negative	Yes, negative	Yes, negative
Serum LDH, U/L	ND	380	ND	425	325	ND	ND	ND	ND
Plasma GM-CSF (pg/mL)	<2.55	<2.55	<2.55	<2.55	<2.55	<2.55	<2.55	<2.55	ND
Chest CT scan	Yes	Yes	Yes	Yes	Yes	Yes	Yes	Yes	Yes
Pulmonary function tests (age)	Yes (9 years)	Yes (7 years)	ND	Yes (11 years)	Yes (9 years)	Yes (20 years)	Yes (15 years)	Yes (11 years)	Yes (18 years)
FVC, % predicted	97	80	?	58.8	74.2	66	51	81	86
FEV1, % predicted	99	57	?	54	65	47	39	79	93
FEV1/FVC, % predicted	100	?	?	95	86	73	77	97	112
TLC, % predicted	97	80	?	?	?	97	126	100	93
VC, % predicted	95	71	?	?	?	71	50	78	102
RV, % predicted	102	?	?	?	?	159	384	187	129
DLCO, % predicted	ND	ND	?	?	?	80	78	116	65
Bronchoscopy and BAL	Yes	Yes	?	Yes	No	?	Yes	?	No
Lung biopsy	Yes	Yes	No	No	No	No	Yes	No	No
Genetic testing – DSP	Yes, negative	Yes, negative	Yes, negative	Yes, negative	Yes, negative	Yes, negative	Yes, negative	Yes, negative	Yes, negative
GM-CSF neutralizing antibody test, result	Yes, negative	Yes, negative	Yes, negative	Yes, negative	Yes, negative	Yes, negative	Yes, negative	Yes, negative	ND
Genetic testing - *CCR2 allele 1, allele 2*	c.640_645del, c.640_645del	c.640_645del, c.640_645del	c.182T>G, c.182T>G	c.887C>A, c.887C>A	c.887C>A, c.887C>A	c.59_60insAC, c.356T>G	c.59_60insAC, c.356T>G	c.59_60insAC, c.356T>G	c.182T>G, c.182T>G

Medical history/ infectious comorbidities	Pneumonia – no micro-organism, COVID-19, recurrent otitis	Pneumonia – RSV, EV, RhV; Pneumonia – *Staphylococcus aureus*, *Stenotrophomo-nas*, COVID-19, recurrent otitis	?	COVID-19, URTI	COVID-19, URTI	Pneumonia – mycoplasma, COVID-19	Pneumonia – no microorganism, COVID-19, Recurrent otitis	COVID-19	febrile seizure, asthma, pilonidal cyst, fibrocystic disease of the breasts, COVID-19
Treatment									
Antibiotics	Yes	Yes	Yes	Yes	Yes	Yes	Yes	Yes	Yes
WLL therapy	No	No	No	No	No	No	No	No	No
Oxygen supplementation	No	No	No	No	No	No	No	No	No
Worst respiratory status (FEV1%)	76	57	?	54	65	47	39	71	93
Clinical outcome	Alive	Alive	Alive	Alive	Alive	Alive	Alive	Alive	Alive
Current age, years	12	8	12	12	10	21	16	14	18
Current respiratory status	DOE	DOE	?	DOE	DOE	DOE	DOE	Asymptomatic	DOE

AE: adverse event; BAL: bronchoalveolar lavage; CT: computed tomography; DOE: dyspnea on exertion; DLCO: diffusion capacity of the lungs for carbon monoxide; DSP: disorders of surfactant production; EV: enterovirus; FEV1: forced expiratory volume in one second; FiO_2: fraction of inspired oxygen; FVC: forced vital capacity; NA: not available; ND: not done; PFTs: pulmonary function tests; RSV: respiratory syncytial virus; RhV: rhinovirus; RV: residual volume; SpO_2: oxygen saturation; TLC: total lung capacity; VC: vital capacity; WLL: whole lung lavage; URTI: upper respiratory tract infection; ?: unknown.

Table S2. Bronchoalveolar lavage cytology for CCR2-deficient patients, related to Figure 6.

Patient	Age (years)	Cells/mL (x10^4)	Macrophages (%)	Lymphocytes (%)	Neutrophils (%)	Eosinophils (%)	BEC (%)	Culture result
Reference[92]	0.25-14	17.92 ± 8.12	93.33 ± 4.56	3.93 ± 3.08	2.94 ± 2.82	NR	NR	Negative
P1	9	12	54	7	38	1	0	Negative
	12	NR	42	NR	48	NR	NR	Negative
P2	7	22	62	17	20	1	0	Negative
	8	18	36	13	50	1	0	Negative
P4	11	40	5	65	30	0	0	Gram$^+$ cocci
P6	10	NR	52	10	35	0	3	Negative
P7	3	NR	67	13	19	1	0	Normal oral flora
	5	NR	68	16	13	3	0	Negative

BEC: bronchial epithelial cells; NR: not reported

Table S3. Homozygous and compound-heterozygous variants identified by WES in eight CCR2-deficient patients, related to Figure 1.

P1	P2	P3	P4	P5	P6	P7	P9
ATRIP, p.R718H	*ATRIP*, p.R718H	*AC004381*.6, p.K725M	*ABCA5*, p.F1102C	*ATN1*, p.Q496_Q502del	*AURKA*, c.42+2ins61	*AURKA*, c.42+2ins61	*ABCB9*, p.G248S
***CCR2*, p.P214_L215del**	*CCDC117*, p.P28L	*ALOXE3*, p.L237M / p.I705T	*ATN1*, p.Q496_Q502del	***CCR2*, p.T296N**	*C20orf132*, c.90+1ins29	*C20orf132*, c.90+1ins29	*ARID1B*, p.S41del / p.A460del
CHPF2, p.A565T	***CCR2*, p.P214_L215del**	*ARHGEF17*, p.Q805E	*C1GALT1C1*, p.H39N	*CKAP5*, p.S1999C	***CCR2*, p.T21Pfs*18 / p.L119R**	***CCR2*, p.T21Pfs*18 / p.L119R**	*ATP1B4*, p.T46M
CPZ, p.D218E / p.R570Q	*CSRNP1*, p.R136W	*BRIP1*, p.Q944E / R1035C	***CCR2*, p.T296N**	*COL21A1*, p.V399A	*FAM58A*, p.G3fs	*FAM58A*, p.G3fs	*ATXN3*, p.G306fs
HHIPL1, p.G77S	*DAK*, p.R567W	*C20orf27*, p.A48T	*CEP128*, p.F842L	*COL7A1*, p.I1597V	*FAM58A*, p.G14fs	*FAM58A*, p.G14fs	*CACTIN*, p.R689C
IPCEF1, p.A411V	*DPYD*, p.A721T / p.D949V	***CCR2*, p.M61R**	*COL15A1*, p.V154G / p.R1387G	*CSMD1*, p.V895M / p.W1348C	*SGPP2*, p.I124L	*SGPP2*, p.I124L	*CCDC86*, p.L11P
KIAA1217, p.R46C / p.K591R	*EOMES*, p.L146F	*CFHR2*, p.R69C	*COL7A1*, p.I1597V	*CYP26B1*, p.V357I			*CCNT1*, p.G3V
LAMB2, p.E628Q	*LAMB2*, p.E628Q	*CFTR*, p.K68E	*CSMD1*, p.V895M / p.W1348C	*DBP*, p.G24S			***CCR2*, p.M61R**
MADCAM, p.P272L / p.S310L	*MADCAM*, p.P272L / p.S310L	*DLEC1*, p.A1555V	*FLNB*, p.T1588M	*DFNB31*, p.V735I / g.117185804C>T			*CTNND1*, p.S905Y
MLYCD, .83945979G>A	*MON1A*, p.T486A	*EEF2K*, p.V185M	*G6PD*, p.S218F	*FABP1*, p.G32R			*DDX58*, p.D701A
MON1A, p.T486A	*NOD2*, p.A918D	*FAM81B*, p.Q285fs	*KCNH8*, p.S86*/p.L549*	*FAM231B*, p.W72fs			*EEA1*, p.P155S
PCSK1, p.P710R	*OTUD3*, p.G47_G48dup	*FAM81B*, p.D287N	*NKTR*, p.L805F	*FAM65C*, p.R239W			*ENDOU*, p.D35Y
PRR12, p.T774S / p.R867H	*PAPPA*, p.R180Q / p.R1087Q	*KCNN3*, p.Q78_Q80dup	*NUP210L*, p.S106G / p.Q1780R	*FLNB*, p.T1588M			*ENOSF1*, p.V406fs
SIGLEC1, p.Q263R	*PRR12*, p.T774S / p.R867H	*LGALS12*, p.G152*	*PAGE1*, p.E138G	*FRMPD3*, p.R659H			*FBLN2*, p.E123K
TOPAZ1, p.R683W	*SIGLEC1*, p.Q263R	*NEK4* g.52785947C>G	*PLCH2*, p.D23Y	*G6PD*, p.S218F			*HRCT1*, p.H102_H105dup
WDR60, p.E334K	*TAOK2*, p.V1129I	*OLFM3*, p.T24I / p.I111V	*RP11-1220K2.2*, p.S2197C / p.T2291I	*GLE1*, p.P2R			*JSRP1*, p.V223I
WDR86, p.R229Q	*TEKT1*, p.E68K	*OR3A1*, p.F34I	*SULT1C2*, p.Y128H	*GMIP*, p.G92R			*KIF26A*, p.K1450E
ZNF106, p.F213V / p.R926G	*TOPAZ1*, p.R683W	*OR5B12*, p.R232C	*WEE1*, p.L302V	*HRC*, p.H643Y			*NCKIPSD*, p.P625L
ZNF208, p.P228_K225del / p.H795R	*UNC93B1*, p.L129I / p.P209L	*PHF8*, p.E747K	*ZKSCAN7*, p.H601P	*LILRA2*, p.R165H / p.A307T			*NEO1*, p.T435R
	ZNF106, p.F213V / p.R926G	*PPAN*, p.E606K / p.P637L	*ZNF195*, p.A24T	*MYO9B*, p.R1148H			*ODF3L2*, p.A242T
		RNASEH2C, p.D115Y	*ZNF417*, p.H40fs	*NKTR*, p.L805F			*OGFOD3*, p.G237R
		SAPCD2, p.R253C / p.G289fs	*ZNF438*, p.Q520E / p.I810S	*NTN1*, p.M210V			*OR5J2*, p.A125V
		SGSM3, p.Q637*		*NUP210L*, p.S106G / p.Q1780R			*PEX1*, p.E379D / p.D1168H

		STAB1, p.T2395I		NYX, p.G355R			*PRKDC*, p.P764L / p.A1237T
		TBC1D2, p.E905K		*OPHN1,* p.A688S			*RING1*, p.A195T
		TMEM123, p.T125I		*OR7A5,* p.S229fs			*RPS6KA5*, p.Asp554Asn
		TOB2, p.S334N		*PIK3R6,* p.A736V			*SLC25A2*, p.A300T
		TTC14, p.S697G		*RABEPK,* p.P135fs			*SLC9B1P1*, p.I74N / p.K222*
		UQCRC1, p.E435K		*RP11-1220K2.2*, p.S2197C / p.T2291I			*SMPD1*, p.L47_A48del / p.V114M
		UTRN, p.E550D / p.T1473M		*WEE1,* p.L302V			*SPDEF*, p.R167Q
		VSIG4, p.M159L		*ZDHHC5,* p.R182H			*TYRO3*, c.1252+2T>C / c.1484-1G>T
		XYLT1, p.R694H		*ZKSCAN7,* p.H601P			*ZNF417*, p.H40fs
		ZNF711, p.G109A		*ZNF195,* p.A24T			
				ZNF417, p.H40fs			
				ZP1, p. D107N			

Table S4. Levels of pro-inflammatory cytokines and chemokines in broncho-alveolar lavage samples of healthy controls (*n* = 12) and two CCR2-deficient patients, related to Figure 4.

Cytokines/chemokines (pg/mL)	Controls	P1	P2
BLC	2.73 ± 1.60	2.64	2.4
CCL-1	136.52 ± 144.92	463.32	331.21
CCL-2	21.64 ± 23.15	251.86	493.33
CCL-3	10.46 ± 24.57	62.08	N.D.
CCL-4	0.64 ± 0.4	2.106	0.547
CCL-7	7.06 ± 2.46	3.82	10.07
CCL-8	0.30 ± 0.12	0.63	2.49
CCL-11	N.D.	N.D.	N.D.
CCL-13	1.16 ± 0.57	8.25	85.346
CCL-17	1.97 ± 1.64	2.38	1.47
CCL-19	1.56 ± 1.49	N.D.	5.13
CCL-20	20.43 ± 16.22	7.87	12.46
CCL-22	1.91 ± 3.34	17.31	111.71
CCL-24	407.69 ± 281.78	327.78	393.41
CXCL-1	990.68 ± 1033.90	2818.28	1168.99
CXCL-2	58.08 ± 71.95	48.13	61.74
CXCL-5	3.19 ± 7.93	N.D.	180.31
CXCL-9	316.41 ± 268.44	320.88	411.24
CXCL-10	58.00 ± 29.39	89.82	80.92
CXCL-11	0.40 ± 0.38	0.163	0.357
CXCL-12	33.66 ± 27.03	144.54	22.29
CX3CL-1	29.48 ± 80.65	N.D	71.6
IFN-α2	0.12 ± 0.45	N.D.	N.D.
IFN-γ	0.26 ± 0.95	7.905	N.D.
IL-1β	15.75 ± 41.44	35.82	N.D.
IL-6	42.56 ± 49.84	63.05	30.96
IL-8	106.62 ± 71.28	872.71	258.09
IL-10	1.92 ± 1.00	1.17	N.D.
IL-12p70	1.91 ± 1.60	1.32	1.01
IL-17A	3.70 ± 6.05	0.301	N.D.
IL-18	15.37 ± 21.95	161.08	16.47
IL-23	N.D.	N.D.	N.D.
IL-33	21.21 ± 23.46	N.D.	N.D.
PTX3	21.33 ± 7.33	95.55	164.32
sCD25	93.65 ± 90.48	172.64	59.28
sCD40L	173.05 ± 238.50	296.76	N.D.
sRAGE	1354.17 ± 2284.87	1111.04	1125.93
sST2	230.22 ± 85.61	303.3	194.9
sTNF-RI	110.71 ± 54.89	328.9	361.73
sTNF-RII	5.61 ± 2.81	5.36	5.8
sTREM-1	42.70 ± 26.25	80.54	85.86
TGF-β1	1.23 ± 4.24	N.D	N.D
TNF	3.86 ± 3.45	N.D.	N.D.

Mean ± SD. N.D. not detected

Table S5. Cell counts in bone-marrow aspirate from patients with CCR2 deficiency, related to Figure 5.

Hematopoietic lineage / cell type	P1	P2	Reference Value
Erythroblastic lineage, % of cells			
Proerythroblasts	1	1	0.5-3
Basophilic erythroblasts	1	2	1-5
Polychromatophilic erythroblasts	3	6	7-20
Acidophilic erythroblasts	8	7	4-15
Granulocytic lineage, % of cells			
Myeloblasts	1	1	0.5-5
Promyelocytes	1	1	1-6
Myelocytes	11	9	8-20
Metamyelocytes	17	16	10-23
Polymorphonuclear neutrophils	30	34	30-70
Eosinophils	0	0	0.5-4
Basophils	0	0	0-1
Lymphoid lineage, % of cells			
Lymphocytes	25	22	3-18
Lymphoblasts	0	0	-
Monocytic lineage, % of cells			
Monoblasts	0	0	-
Promonocytes	0	0	-
Monocytes	2	1	0-5
Others	0	0	-
