# Supplementary material for: Human inherited CCR2 deficiency underlies progressive polycystic lung disease
Source: Cell. Author manuscript; Available in PMC 2024 Feb 5. (PMC10842692; doi:10.1016/j.cell.2023.11.036)
Supplement: 1 [file NIHMS1949155-supplement-1.pdf]

Supplemental figures

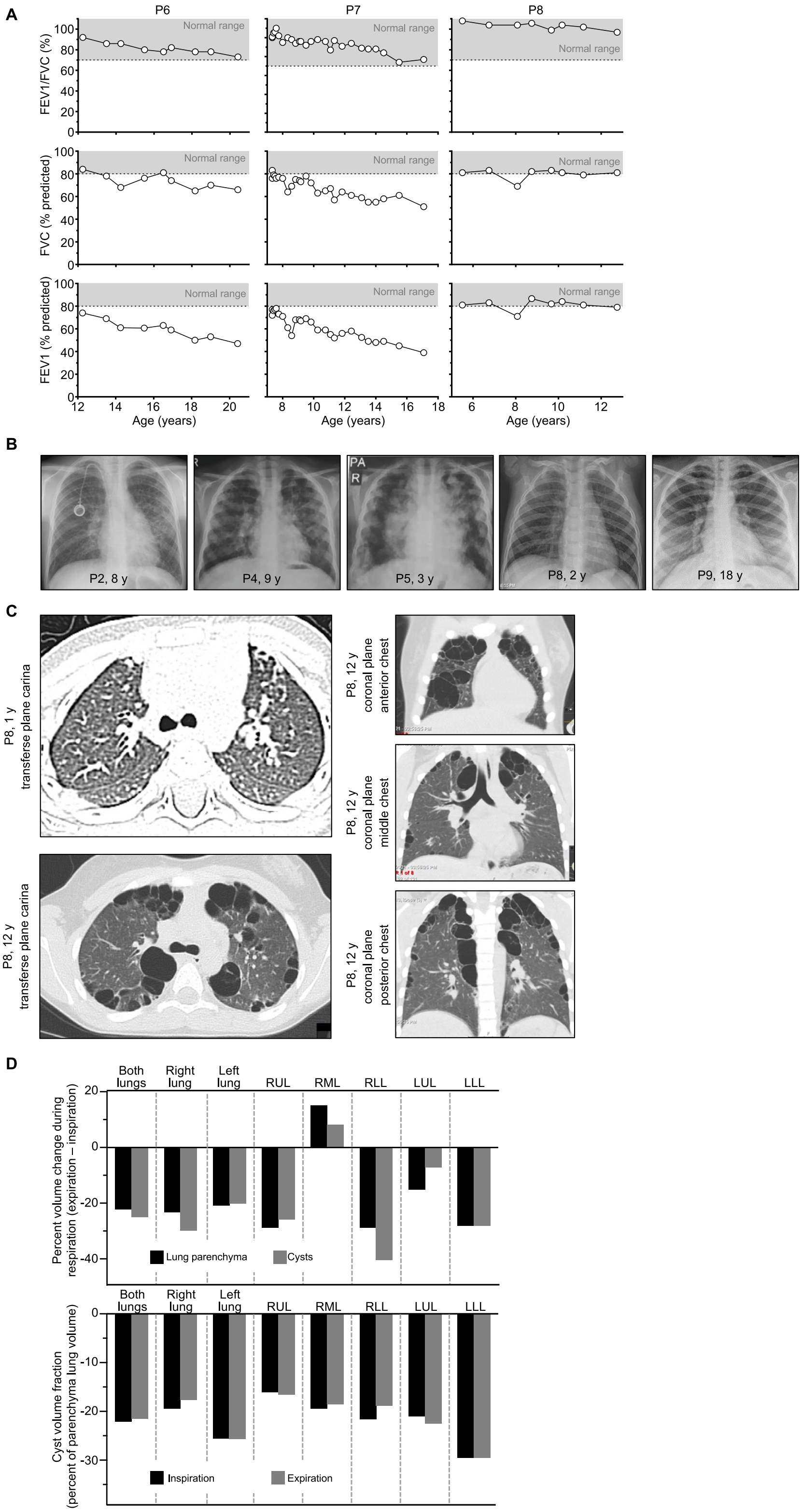

(legend on next page)

Figure S1. Chest radiography, pulmonary function, and quantitative lung cyst volume evaluations for the CCR2-deficient patients, related to Figure 1

(A) Serial pulmonary function test results (P6–P8) at the ages indicated, showing oxygen saturation (SpO_2), forced expiratory volume in 1 second (FEV1), forced vital capacity (FVC), and residual volume (RV) as a percentage of the predicted value.

(B) Posterior-anterior chest X-rays for P2, P4, P5, P8, and P9, respectively, at the ages indicated.

(C) Chest CT images of P8 at the ages indicates.

(D) Quantitative volumetric measurement of the relative change in the regional volume of lung cysts and normal lung (left panel) and cyst volume fraction (right panel) during respiration in a CCR2-deficient patient (P6). RUL, right upper lobe; RML, right middle lobe; RLL, right lower lobe; LUL, left upper lobe; LLL, left lower lobe.

CellPress
OPEN ACCESS

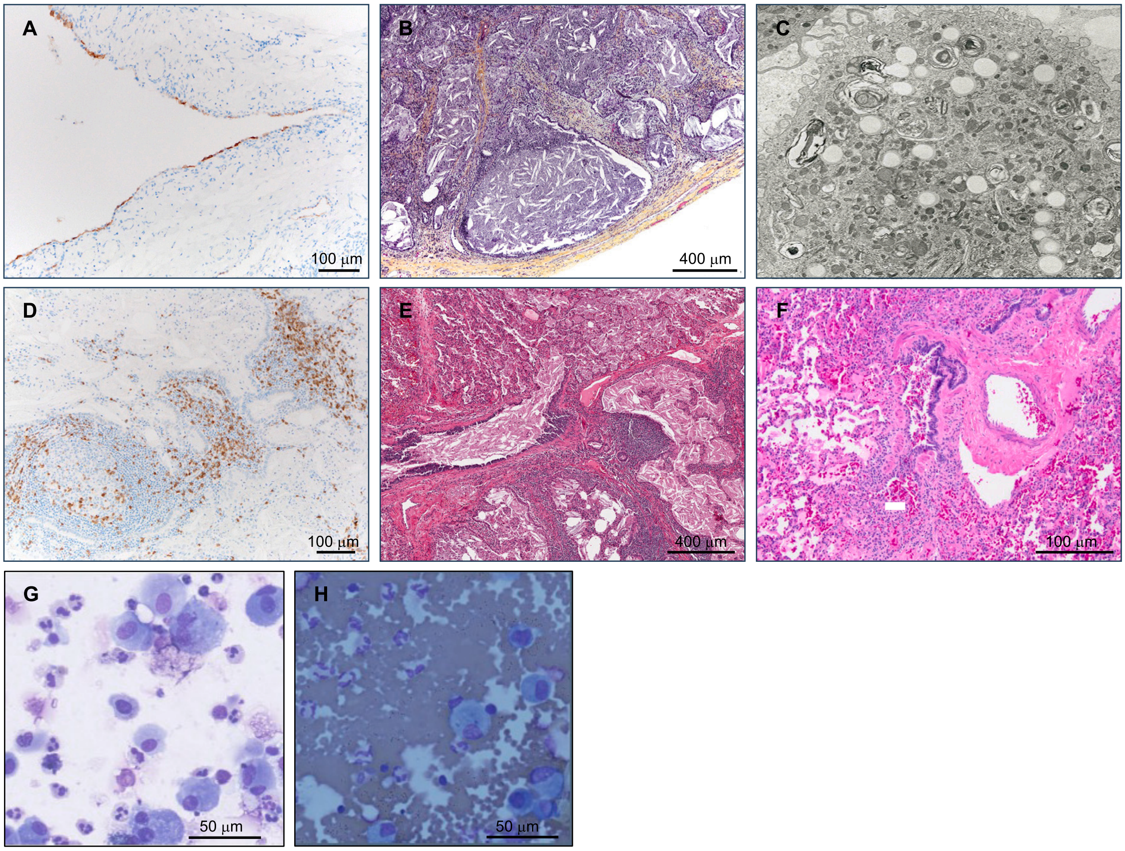

Figure S2. Lung histology and bronchoalveolar lavage cytology, related to Figure 1

(A–F) Surgical lung biopsy specimen from a CCR2-deficient patient (P7) at the age of 3 years, showing aquaporin expression in sessile epithelial cells lining cysts (aquaporin immunostain) (A), cysts adjacent to the pleural surface thickened by fibrosis resulting in honeycombing with parenchymal cyst cavities filled with PAP sediment and cholesterol clefts (pentachrome staining) (B), electron microscopy of alveolar type 2 epithelial cells showing normal lamellar bodies (uranyl acetate) (C), T cells present in peribronchiolar follicular lymphocytosis (CD3 immunostain) (D), a terminal bronchiole distorted by peribronchiolar fibrosis and extensive follicular lymphocytosis contiguous with a cyst filled with eosinophilic granular sediment and cholesterol crystals (H&E) (E), and a terminal bronchiole distorted by peribronchiolar and subepithelial fibrosis and lymphocytic bronchiolitis (F).

(G and H) Bronchoalveolar lavage from CCR2-deficient patients: P1 at the age of 12 years (G) and P2 at the age of 8 years (H). Note that the alveolar macrophages are not grossly enlarged and foamy appearing.

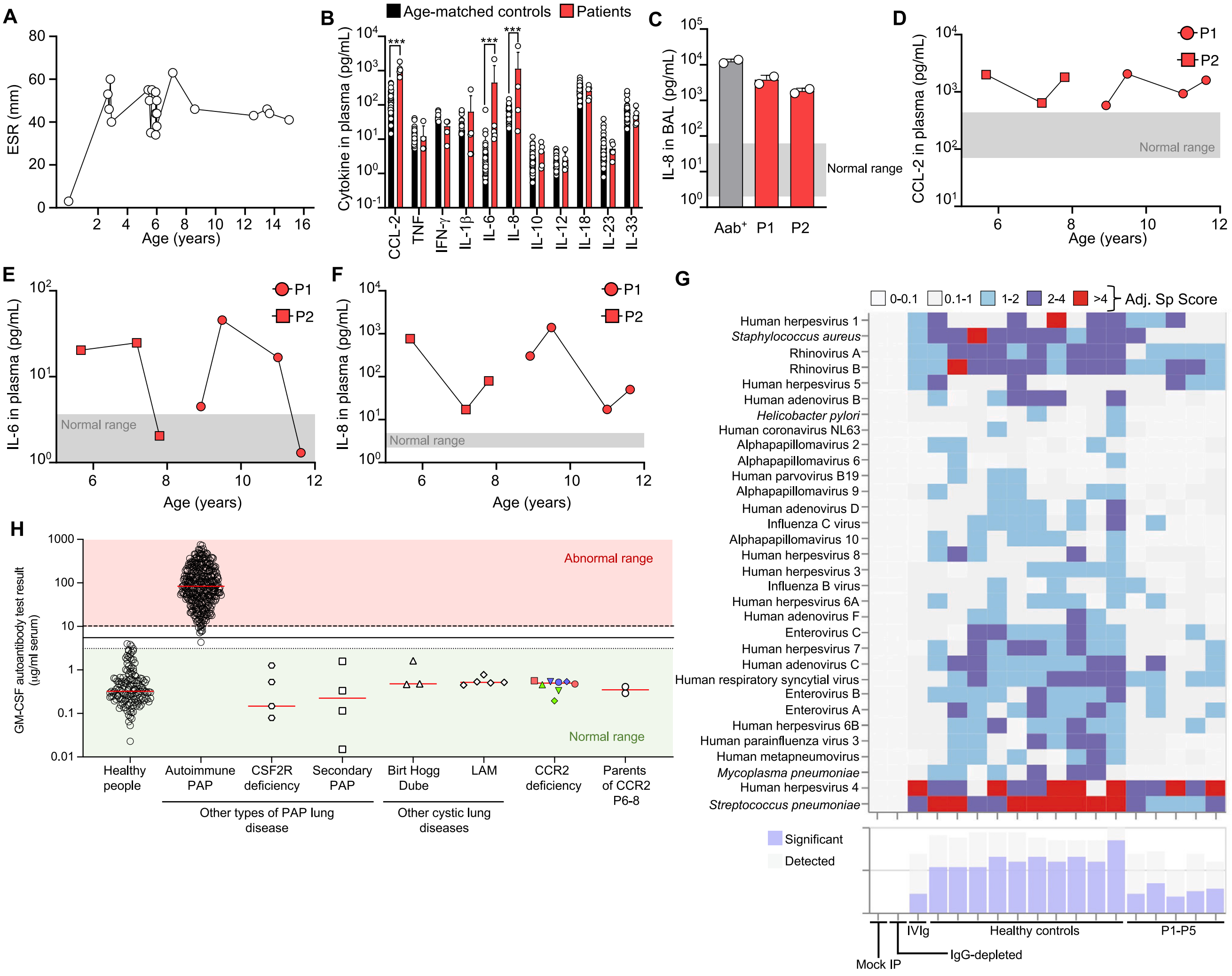

Figure S3. Laboratory evidence of pulmonary and systemic inflammation, anti-GM-CSF autoantibody testing, and adaptive immune responses in patients with CCR2 deficiency, related to Figure 2

(A) Natural course of serial erythrocyte sedimentation rate (ESR) measurements in P7.

(B) Concentration of selected cytokines, measured by ELISA in plasma samples from P1 to P5 and in age-matched healthy controls (n = 51).

(C) IL-8 levels in bronchoalveolar lavage (BAL) from P1 and P2 and a patient with autoimmune PAP (Aab$^+$).

(D–F) Concentration of (D) CCL-2, (E) IL-6, and (F) IL-8 in plasma from P1 and P2 at the ages indicated.

(G) Heatmap depicting score values equivalent to the counts of peptides displaying significant enrichment in plasma samples from pediatric controls (n = 10) and P1–P5. Pooled plasma used for intravenous immunoglobin (IVIg) therapy and IgG-depleted serum were used as positive and negative controls, respectively.

(H) Detection of GM-CSF autoantibodies in plasma and serum samples from healthy controls, patients with pulmonary alveolar proteinosis (PAP), other cystic lung diseases (BHD, LAM), the patients with CCR2 deficiency (P1–P8) and the healthy parents of P6–P8. BHD, Birt Hogg Dube; LAM, lymphangioleiomyomatosis. For (B) and (H) the data shown are the mean ± SD, for C, the data shown are the means of technical duplicates ± SD. Significance was assessed using one-way ANOVA with Turkey's test for multiple comparison (B); ***p ≤ 0.001.

Figure S4

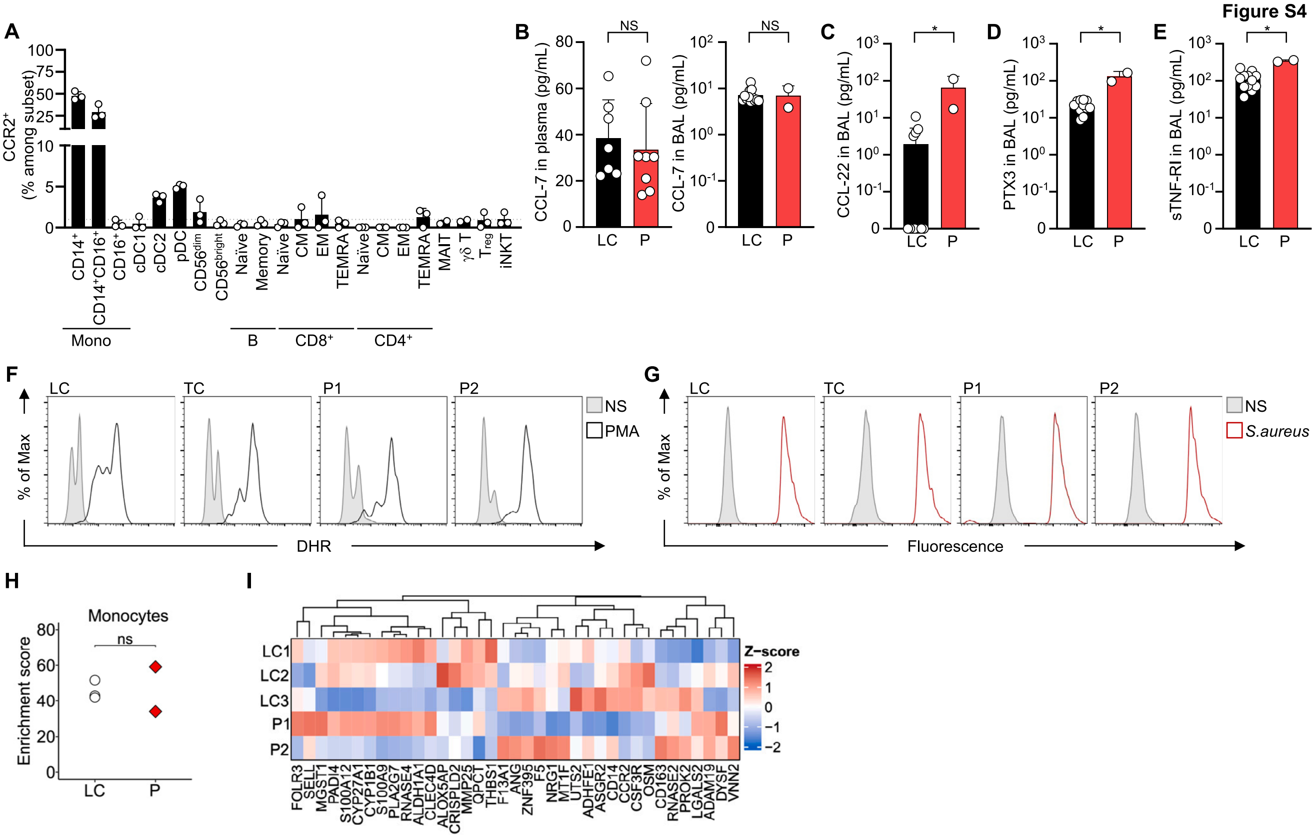

Figure S4. Expression of CCR2, impact of CCR2 deficiency on monocyte function, and expression of inflammatory markers, related to Figure 4

(A) Percentage of cells expressing CCR2 for the various subsets of peripheral hematopoietic cells from healthy controls (n = 3), as determined by conventional flow cytometry staining. cDCs, classical dendritic cells; pDCs, plasmacytoid dendritic cells; CM, central memory; EM, effector memory; TEMRA, effector memory re-expressing CD45RA; MAIT, mucosal-associated invariant T cells; T_{reg}, regulatory T cells; iNKT, invariant natural killer T cells.

(B) CCL-7 levels in the plasma of P1–P8 and controls (LC; n = 5) and the bronchoalveolar lavage (BAL) of controls (n = 12), P1, and P2.

(C–E) Concentration of (C) CCL-22, (D) PTX3, and (E) sTNFR-RI in BAL samples from controls (n = 12), P1, and P2.

(F) Intracellular ROS production measured by DHR in monocytes from local (LC) and travel (TC) controls, P1, and P2 upon PMA stimulation.

(G) Phagocytosis of pHrodo *S. aureus* bioparticles by CD14+ monocytes from controls, P1, and P2.

(H and I) Enrichment score (H) and heatmap (I) for genes defining classical monocytes expressed in controls, P1, and P2, as determined by RNA sequencing performed on sorted CD14+ monocytes. For (A)–(E), the data shown are the mean ± SD. Significance was assessed using Mann-Whitney U tests (B–E); ns, not significant, *$p \leq 0.05$.

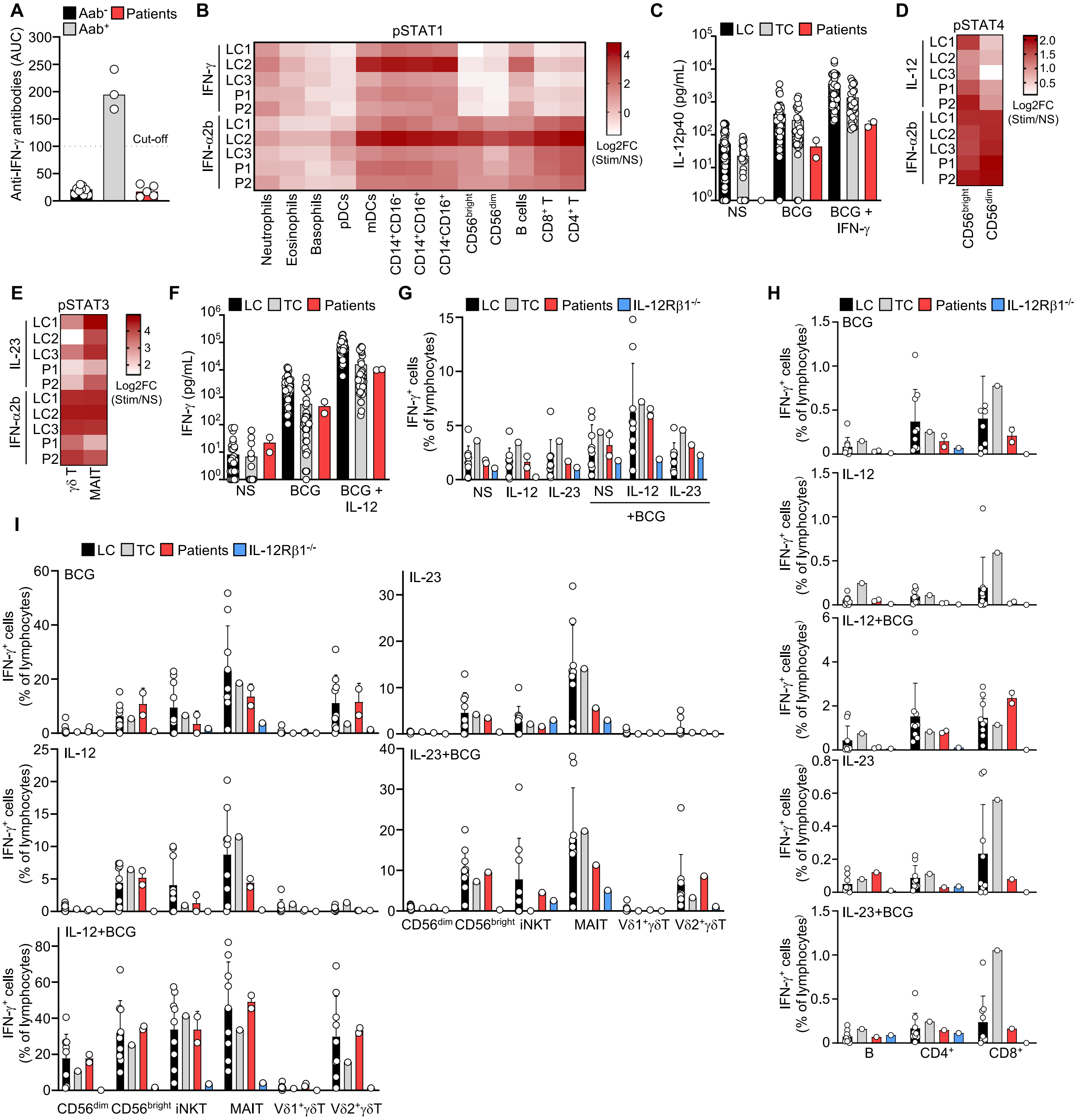

Figure S5. Conserved IFN-γ-related immunity in the cells of the CCR2-deficient patients *in vitro*, related to Figure 4

(A) Detection of anti-IFN-γ autoantibodies in plasma samples from healthy controls (Aab⁻, n = 9), patients known to have anti-IFN-γ autoantibodies (n = 3, Aab⁺), and P1–P5.

(B) CyTOF analysis for phospho-STAT1 (pSTAT1) on whole blood from healthy local controls (n = 3), P1, and P2 after IFN-γ (1,000 IU/mL) or IFN-α2b (1,000 IU/mL) stimulation. mDCs, myeloid dendritic cells; pDCs, plasmacytoid dendritic cells; FC, fold change.

(C) Secretion of IL-12p40 in whole blood from local (LC; n = 62) and travel (TC; n = 32) controls, P1, and P2, with and without stimulation with BCG (MOI 20) or BCG and IFN-γ (1,000 IU/mL).

(D and E) CyTOF analysis for (D) pSTAT4 after IL-12 (20 ng/mL) and IFN-α2b (1,000 IU/mL) stimulation in NK cells (CD56dim, CD56bright) or (E) pSTAT3 after IL-23 (100 ng/mL) and IFN-α2b (1,000 IU/mL) stimulation in γδ T and mucosal-associated invariant T cells (MAIT) from healthy controls, P1, and P2.

(F) Secretion of IFN-γ in whole blood from controls (LC, n = 47; TC, n = 40), P1, and P2, with and without stimulation with BCG (MOI 20) or BCG and IL-12 (20 ng/mL).

(legend continued on next page)

(G–I) Intracellular production of IFN-γ by (G) total PBMCs; (H) B cells, CD4$^+$ T cells, and CD8$^+$ T cells; (I) NK cells, invariant natural killer T cells (iNKT), MAIT, and γδ T cells from controls (LC, n = 9; TC = 1); P1; P2; and an IL12Rβ1-deficient patient with and without stimulation with IL-12 (20 ng/mL), IL-23 (100 ng/mL), BCG (MOI of 20), BCG + IL-12, and BCG + IL-23, as assessed by intracellular flow cytometry. Stimulation with IL-23 and BCG + IL-23 was performed only on P1's PBMCs. For (A), (C), and (F)–(I), the data shown are mean ± SD.

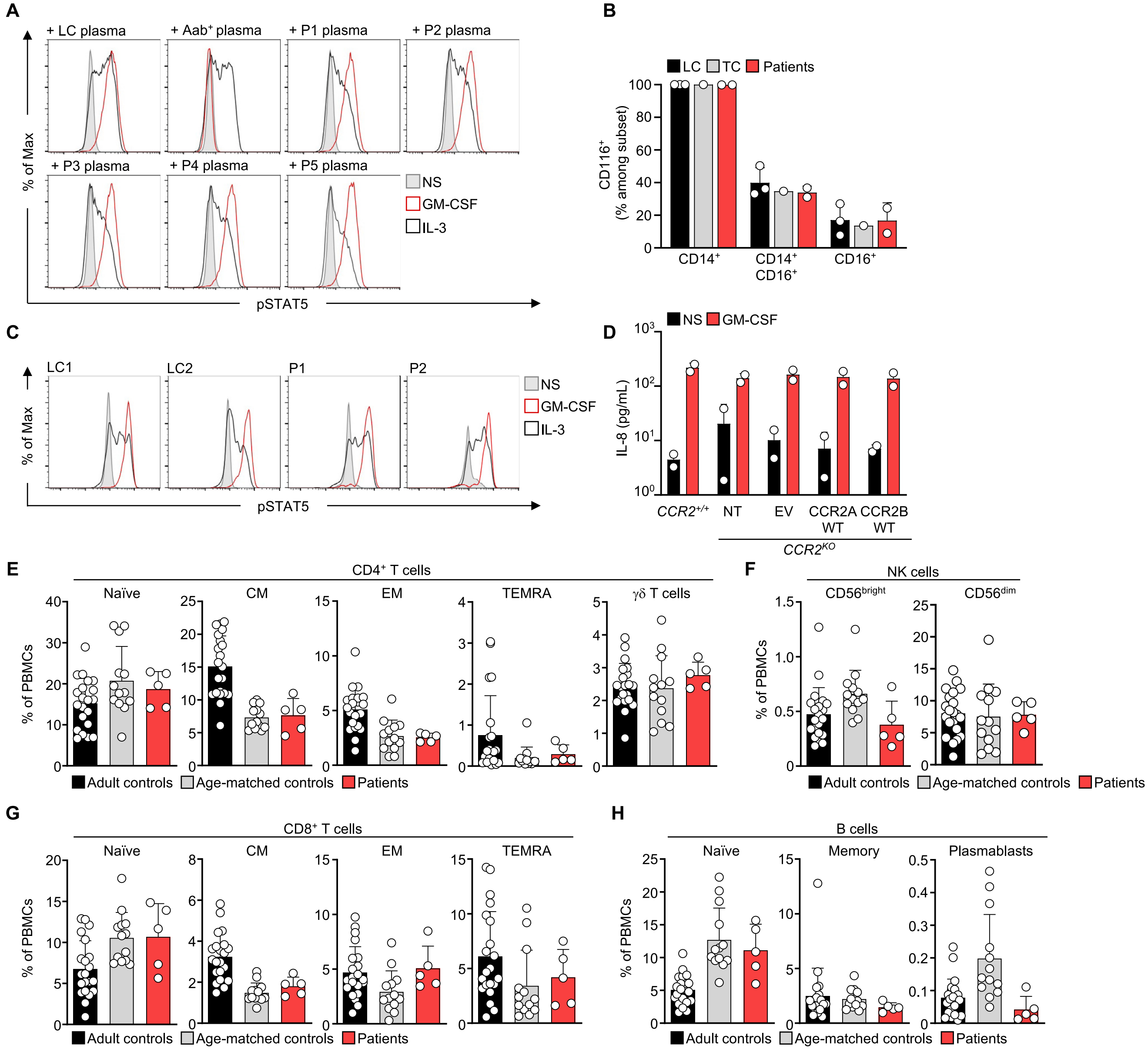

Figure S6. GM-CSF signaling in CCR2-deficient cells and immunophenotyping of CCR2-deficient patients, related to Figure 5

(A) FACS plots depicting the amounts of pSTAT5 induced by GM-CSF (20 ng/mL) or IL-3 (100 ng/mL) in healthy control cells (gated on CD14^{+} monocytes) in the presence of 10% plasma from a healthy control (LC), a patient known to have neutralizing autoantibodies against GM-CSF (Aab^{+}), or plasma from P1 to P5.

(B) Quantification of CD116 expression on the monocyte subsets of P1–P5 and local (LC, n = 3) and travel (TC, n = 1) controls.

(C) FACS plots depicting the amounts of pSTAT5 induced by GM-CSF (20 ng/mL) or IL-3 (100 ng/mL) in CD14^{+} monocytes from healthy controls, P1, and P2.

(D) GM-CSF (100 ng/mL)-induced IL-8 secretion by THP-1 CCR2^{KO} cells without transduction (NT) or transduced with an empty vector (EV) or wild-type (WT) *CCR2A* or *CCR2B*.

(E–H) Frequency of (E) CD4^{+} T cell subsets and (F) NK cell subsets and (G) CD8^{+} T cell subsets and (H) B cell subsets in adult controls (n = 21), age-matched controls (n = 13), and P1–P5, as determined by CyTOF. CM, central memory; EM, effector memory; TEMRA, effector memory re-expressing CD45RA. For (B) and (D)–(H), the data are mean ± SD.

CellPress
OPEN ACCESS

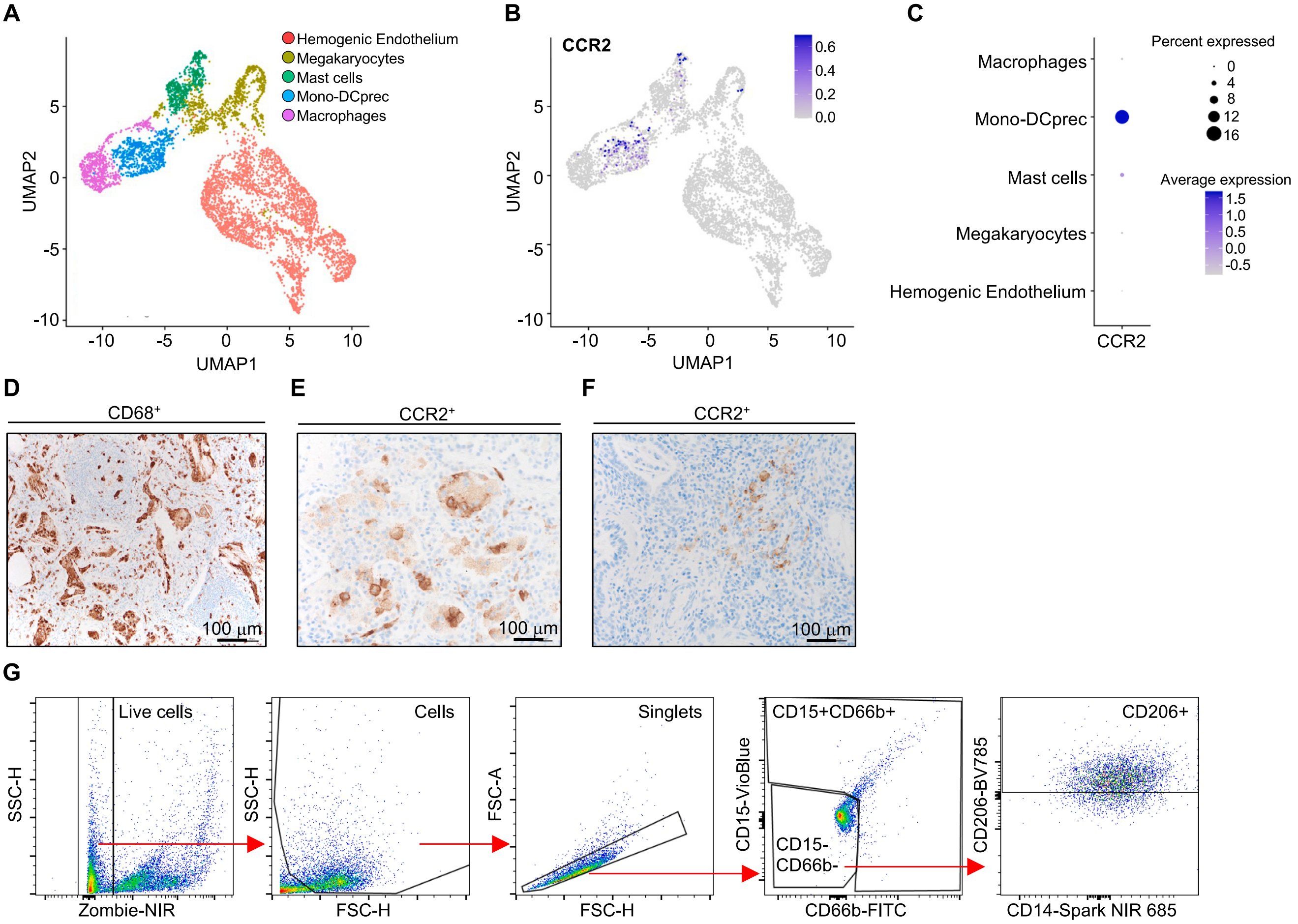

Figure S7. Analysis of lung biopsies and bronchoalveolar lavage samples, related to Figure 6

(A) UMAP projections of single cells from hematopoietic iPSC-derived organoids. Cluster annotations were predicted using logistic regression classifiers trained on publicly available data.[54]

(B) CCR2 expression levels projected onto UMAP.

(C) Gene expression of *CCR2* in predicted cell types.

(D–F) Surgical lung biopsy specimen from a patient with PAP caused by *SFTPC* mutations, with immunostaining to identify CD68$^+$ macrophages (D) or CCR2$^+$ cells (E). (F) Surgical lung biopsy from a patient with unspecified interstitial lung disease showing CCR2-positive cells (brown immunostaining for CCR2).

(G) Gating strategy for flow cytometric analysis of cryo-preserved bronchoalveolar lavage samples.
